# Set1 and Kdm5 are antagonists for H3K4 methylation and regulators of the major conidiation‐specific transcription factor gene *ABA1* in *Fusarium fujikuroi*

**DOI:** 10.1111/1462-2920.14339

**Published:** 2018-09-18

**Authors:** Slavica Janevska, Ulrich Güldener, Michael Sulyok, Bettina Tudzynski, Lena Studt

**Affiliations:** ^1^ Institute of Plant Biology and Biotechnology Westfälische Wilhelms‐Universität Münster Münster Germany; ^2^ Department of Bioinformatics TUM School of Life Sciences Weihenstephan, Technical University of Munich Freising Germany; ^3^ Center for Analytical Chemistry, Department IFA‐Tulln University of Natural Resources and Life Sciences Vienna Austria; ^4^ Department of Applied Genetics and Cell Biology‐Tulln, University of Natural Resources and Life Sciences Vienna Austria

## Abstract

Here we present the identification and characterization of the H3K4‐specific histone methyltransferase Set1 and its counterpart, the Jumonji C demethylase Kdm5, in the rice pathogen *Fusarium fujikuroi*. While Set1 is responsible for all detectable H3K4me2/me3 in this fungus, Kdm5 antagonizes the H3K4me3 mark. Notably, deletion of both *SET1* and *KDM5* mainly resulted in the upregulation of genome‐wide transcription, also affecting a large set of secondary metabolite (SM) key genes. Although H3K4 methylation is a hallmark of actively transcribed euchromatin, several SM gene clusters located in subtelomeric regions were affected by Set1 and Kdm5. While the regulation of many of them is likely indirect, H3K4me2 levels at gibberellic acid (GA) genes correlated with GA biosynthesis in the wild type, Δ*kdm5* and OE::*KDM5* under inducing conditions. Whereas Δ*set1* showed an abolished GA_3_ production in axenic culture, phytohormone biosynthesis was induced *in planta*, so that residual amounts of GA_3_ were detected during rice infection. Accordingly, Δ*set1* exhibited a strongly attenuated, though not abolished, virulence on rice. Apart from regulating secondary metabolism, Set1 and Kdm5 function as activator and repressor of conidiation respectively. They antagonistically regulate H3K4me3 levels and expression of the major conidiation‐specific transcription factor gene *ABA1* in *F. fujikuroi*.

## Introduction

*Fusarium fujikuroi* is a phytopathogenic ascomycete and the founding member of the *Fusarium* (*Gibberella*) *fujikuroi* species complex (Nirenberg and O'Donnell, 1998; Leslie and Summerell, 2006). *F. fujikuroi* is the causative agent of the so‐called *bakanae* (foolish‐seedling) disease of rice plants which was first described over 100 years ago in Japan (Hori, 1890). Since then, the fungus has gained considerable attention due to the fact that it is a major threat to rice‐growing countries worldwide. Its secretion of the bioactive phytohormones gibberellic acids (GAs) results in thin, chlorotic and hyper‐elongated rice internodes, oftentimes sterile grains, and therefore full harvest losses (Sun and Snyder, 1981; Bömke and Tudzynski, 2009). On the contrary, *F. fujikuroi* is exploited as efficient producer of GAs for the use as plant growth regulators in agriculture and horticulture (Rademacher, 1997; Sponsel and Hedden, 2004).

GAs belong to the group of secondary metabolites (SMs) that are per definition not required for fungal growth in general, but might pose an advantage to the fungus under certain environmental conditions (Fox and Howlett, 2008). Recent sequencing of the *F. fujikuroi* genome revealed 47 predicted SM key genes (Wiemann *et al*., 2013). Next to the GA gene cluster, our group has identified several additional SMs and the respective biosynthetic genes in *F. fujikuroi* over the last years (Janevska and Tudzynski, 2018). Among those are the red pigments bikaverin (BIK) and fusarubins (FSR), two polyketide synthase (PKS)‐derived SMs (Wiemann *et al*., 2009; Studt *et al*., 2012). The mycotoxins apicidin F (APF) and beauvericin are produced by non‐ribosomal peptide synthetases (NRPSs) (Niehaus *et al*., 2014a, 2016), while two other mycotoxins, fusarins (FUS) and fusaric acid (FSA), are generated by a hybrid PKS‐NRPS enzyme and by two separate key enzymes respectively (Niehaus *et al*., 2013, 2014b; Studt *et al*., 2016a).

Fungal secondary metabolism is regulated by a complex network functioning on different hierarchical levels, including so‐called pathway‐/cluster‐specific transcription factors (TFs) that are often encoded within the respective gene cluster (Brakhage, 2013), as well as global regulators. The latter are TFs which affect a larger set of genes of both primary and secondary metabolism in response to different environmental cues, such as the availability of carbon and nitrogen sources or the presence of certain pH and light conditions (Macheleidt *et al*., 2016). On a higher level of regulation, SM biosynthesis underlies the controlled change of the chromatin state which can be either tightly condensed (i.e., heterochromatin) or more loosely packed (i.e., euchromatin), depending on the specific set of post‐translational histone modifications deposited in response to environmental cues (Gacek and Strauss, 2012).

Nearly all of the identified histone (H) modifications are reversible and dynamic. The acetylation of lysine (K) residues is associated with an active transcription, while the methylation of lysine or arginine residues gives a more complex output, depending on associated reader proteins (Brosch *et al*., 2008; Gacek and Strauss, 2012). In general, the methylation of H3K9 and H3K27 is involved in the formation of constitutive and facultative heterochromatin, respectively (Rando and Chang, 2009; Wiles and Selker, 2017), regions predominantly found at centromeres and subtelomeres in *F. fujikuroi* (Wiemann *et al*., 2013; Studt *et al*., 2016b). In contrast, methylation of H3K4 and H3K36 is considered as hallmark of euchromatin in yeast and higher eukaryotes (Rando and Chang, 2009; Wagner and Carpenter, 2012). However, the picture seems to be more diverse in filamentous fungi, as recent data revealed the ubiquitous presence of the H3K36 trimethylation (me3) mark and accordingly, no correlation with active transcription in both *F. fujikuroi* and *Fusarium graminearum* (Connolly *et al*., 2013; Janevska *et al*., 2018).

H3K4 methylation has been indeed found in transcribed euchromatic regions in *F. fujikuroi* (Wiemann *et al*., 2013), and has been clearly linked to actively expressed genes in budding yeast and filamentous fungi, that is, *Saccharomyces cerevisiae*, *Aspergillus nidulans* and *F. graminearum* (Pokholok *et al*., 2005; Connolly *et al*., 2013; Gacek‐Matthews *et al*., 2016). In 5′ promoter regions, H3K4 methylation is associated with the initiating form of RNA polymerase II: all euchromatic genes were found to be decorated with H3K4me2, while H3K4me3 marked actively or recently transcribed genes in *S. cerevisiae* (Santos‐Rosa *et al*., 2002; Ng *et al*., 2003). Noteworthy, *S. cerevisiae* chromatin does not harbour the repressive marks H3K9me3 and H3K27me3, and H3K4 methylation has been implicated in the silencing of subtelomeric genes (Briggs *et al*., 2001; Krogan *et al*., 2002; Bryk *et al*., 2002; Fingerman *et al*., 2005). However, the exact mechanism remains to be elucidated.

The methylation of H3K4 has been studied in a number of different organisms from yeast to humans, and has been shown to depend on the conserved Su(var)3‐9, Enhancer‐of‐zeste, Trithorax (SET) domain‐containing methyltransferase Set1 (Briggs *et al*., 2001; Lee and Skalnik, 2005; Freitag, 2017). The genomes of yeast and filamentous fungi, such as *A. nidulans*, *F. graminearum* and *Fusarium verticillioides*, encode one Set1 homologue that is responsible for all three methylation states H3K4me1/me2/me3 (Briggs *et al*., 2001; Fingerman *et al*., 2005; Harting *et al*., 2013; Govindaraghavan *et al*., 2014; Liu *et al*., 2015; Gu *et al*., 2017). As a counterpart, the Jumonji C (JmjC) domain‐containing demethylase Jhd2 has been identified in *S. cerevisiae* (Liang *et al*., 2007; Seward *et al*., 2007), and later also in *A. nidulans* (Gacek‐Matthews *et al*., 2016). *S. cerevisiae* Jhd2 and the *A. nidulans* homolog KdmB have been shown to counteract H3K4me3 *in vivo* (Liang *et al*., 2007; Gacek‐Matthews *et al*., 2016).

Set1 is part of COMPASS (*Com*plex of *P*roteins *As*sociated with *S*et1). Recently, we identified and functionally characterized the COMPASS component Ccl1 in *F. fujikuroi* and *F. graminearum*. In both fungi, Ccl1 is a critical factor for efficient genome‐wide H3K4 trimethylation by COMPASS and has great impact on SM biosynthesis (Studt *et al*., 2017). However, the exact mechanism of how COMPASS affects the expression of subtelomeric SM genes in both fungi remains unclear so far.

In the present work, we focused on the catalytic subunit Set1 of COMPASS, and on the H3K4me3‐specific demethylase Kdm5 as counterpart of Set1. We found a great impact on genome‐wide transcription, especially upon *SET1* deletion, as well as a considerable effect on SM production for Δ*set1*, Δ*kdm5* and OE::*KDM5* mutants. Notably, albeit completely abolished GA production levels of Δ*set1* in axenic culture, the mutant induced – though strongly attenuated – *bakanae* symptoms on rice. This phenotype goes in line with residual GA_3_ levels found *in planta*. Next to secondary metabolism, Set1 and Kdm5 function as activator and repressor of conidiation respectively. Gene knock‐out, chromatin immunoprecipitation (ChIP) and/or gene expression analyses revealed the conidiation‐specific TF‐encoding gene *ABA1* as major target of Set1, Kdm5 and also other regulators, that is, Csm1, Flb3 and Flb4.

## Results

### *Identification of the H3K4 methyltransferase Set1 and demethylase Kdm5 in* F. fujikuroi

**Figure 1:**
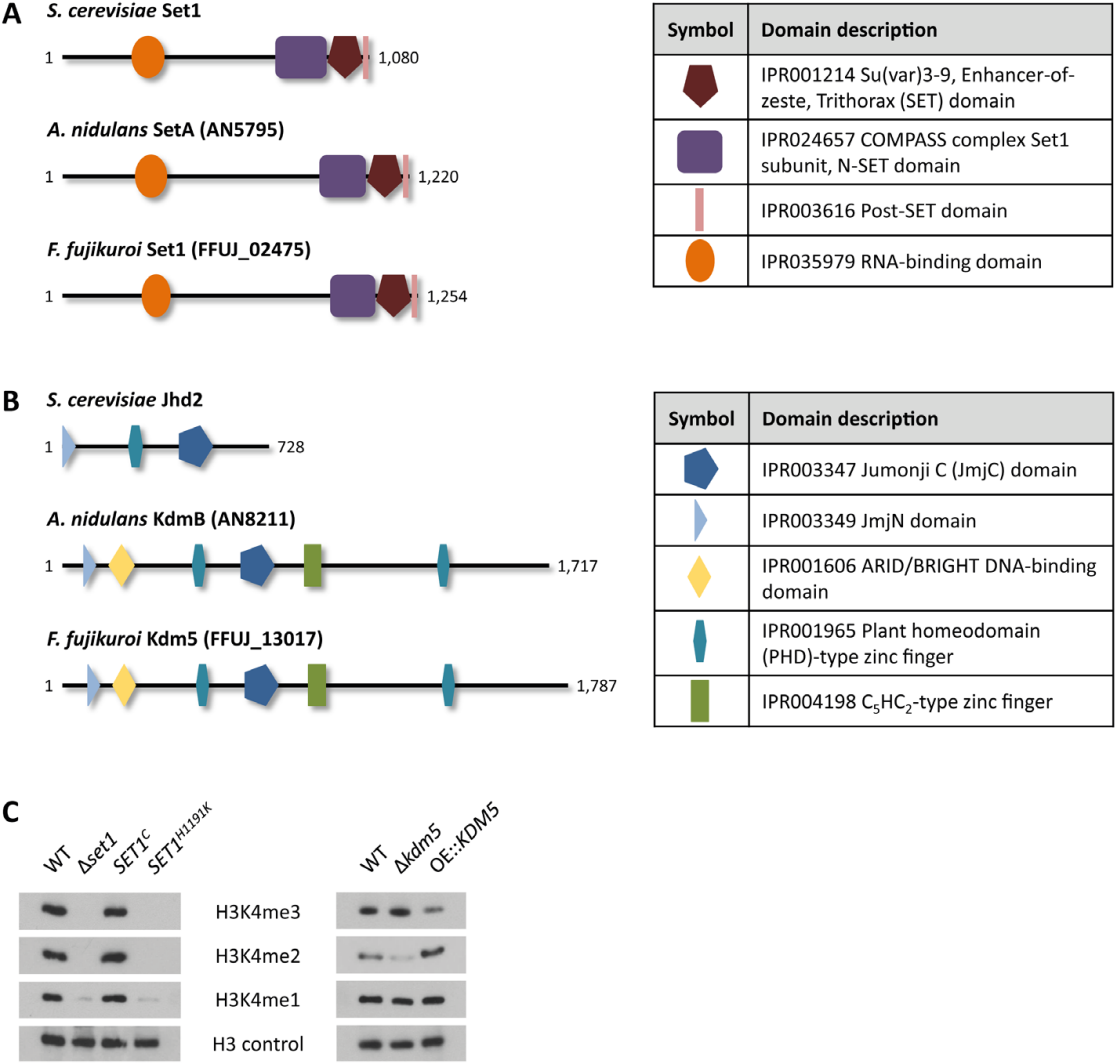
*Fusarium fujikuroi* Set1 and Kdm5 are a H3K4‐specific histone methyltransferase and demethylase respectively. **A,B**. Schematic representation of the domain structure of Set1 and Kdm5 homologues conserved in *S. cerevisiae*, *A. nidulans* and *F. fujikuroi*. The domain description includes the respective InterPro accession numbers. **C**. Western blot analysis using the H3K4me3/me2/me1 and H3 antibodies. Indicated strains were grown in liquid culture (ICI + 60 mM Gln) for 3 days prior to protein extraction. For the detection of H3K4me3 in Δ*kdm5* and OE::*KDM5* mutants, 10 μg of the protein extract was loaded on to the gel, while 15 μg was loaded for the rest.

After analyzing the COMPASS component Ccl1 in *F. fujikuroi* (Studt *et al*., 2017), we have now identified the putative methyltransferase of this complex by determining the ortholog using QuartetS (Yu *et al*., 2011). The predicted protein FFUJ_02475 is the ortholog of the well‐characterized H3K4‐specific methyltransferases Set1 in *S. cerevisiae* and SetA in *A. nidulans* (Briggs *et al*., 2001; Govindaraghavan *et al*., 2014). FFUJ_02475 contains the catalytically active SET (IPR001214), N‐SET (IPR024657) and Post‐SET (IPR003616) domains as well as an RNA‐binding (IPR035979) domain also present in Set1 and SetA from *S. cerevisiae* and *A. nidulans*, respectively (Fig. 1A), and was therefore designated *F. fujikuroi* Set1.

In addition to Set1, FFUJ_13017 was determined by QuartetS as the *A. nidulans* KdmB ortholog (Gacek‐Matthews *et al*., 2016; Yu *et al*., 2011), a putative H3K4‐specific demethylase and possible antagonist to *F. fujikuroi* Set1. Hence, FFUJ_13017 was designated *F. fujikuroi* Kdm5 according to the general nomenclature (Allis *et al*., 2007). Both *F. fujikuroi* Kdm5 and *A. nidulans* KdmB show an identical domain structure which is more complex than that of the *S. cerevisiae* ortholog Jhd2 (Liang *et al*., 2007). All three share the catalytically active JmjC (IPR003347) and JmjN (IPR003349) domains, while KdmB and Kdm5 harbor not only one but two plant homeodomain‐type zinc fingers (IPR001965). In addition, the latter have ARID/BRIGHT DNA‐binding (IPR001606) and C_5_HC_2_‐type zinc finger (IPR004198) domains (Fig. 1B).

Single deletion mutants have been successfully generated for *F. fujikuroi SET1* and *KDM5*, and *KDM5* was additionally overexpressed using the constitutive *oliC* promoter from *A. nidulans*. While abundant in the *F. fujikuroi* wild type (WT) IMI58289, all detectable global H3K4me3 and H3K4me2 signals were lost in Δ*set1* mutants as identified by western blot analyses (Fig. 1C). Contrary to *S. cerevisae* and *A. nidulans* (Fingerman *et al*., 2005; Govindaraghavan *et al*., 2014), but in agreement with *F. graminearum* (Liu *et al*., 2015), a faint band was still detectable for H3K4me1. However, it remains unclear at this moment whether there are indeed residual monomethylation levels in Δ*set1* or whether this band is due to insufficient antibody specificity. The methylation defect was completely restored upon *in loco* complementation of Δ*set1* with the full‐length gene, gaining *SET1* ^*C*^ strains (Fig. 1C). In contrast, the *in loco* integration of the point‐mutated variant *SET1* ^*H1191K*^, in which the catalytically active histidine of the SET domain has been mutated to a lysine residue (Tanaka *et al*., 2007; Janevska *et al*., 2018), did not complement as expected (Fig. 1C).

Next, *F. fujikuroi* Kdm5 was identified as a true counterpart of Set1, functioning as an H3K4me3‐specific demethylase. The total level of H3K4me3 was increased in Δ*kdm5* and decreased in OE::*KDM5*, while the opposite was true for H3K4me2 (Fig. 1C). More precisely, H3K4me3 cannot be removed in the Δ*kdm5* mutant, which results in a shift of H3K4me2 to H3K4me3 genome‐wide (Fig. 1C; Supporting Information Fig. S1), as H3K4me2 is still continuously converted into the trimethylation state by Set1. Something very similar has also been observed for *S. cerevisiae JHD2* and *A. nidulans kdmB* deletion mutants (Liang *et al*., 2007; Gacek‐Matthews *et al*., 2016).

In summary, Set1 is responsible for H3K4 tri‐, di‐ and possibly also monomethylation in *F. fujikuroi*, while Kdm5 was found to counteract Set1‐mediated H3K4me3.

### *Deletion of* SET1 *and overexpression of* KDM5 *affect vegetative growth*

**Figure 2:**
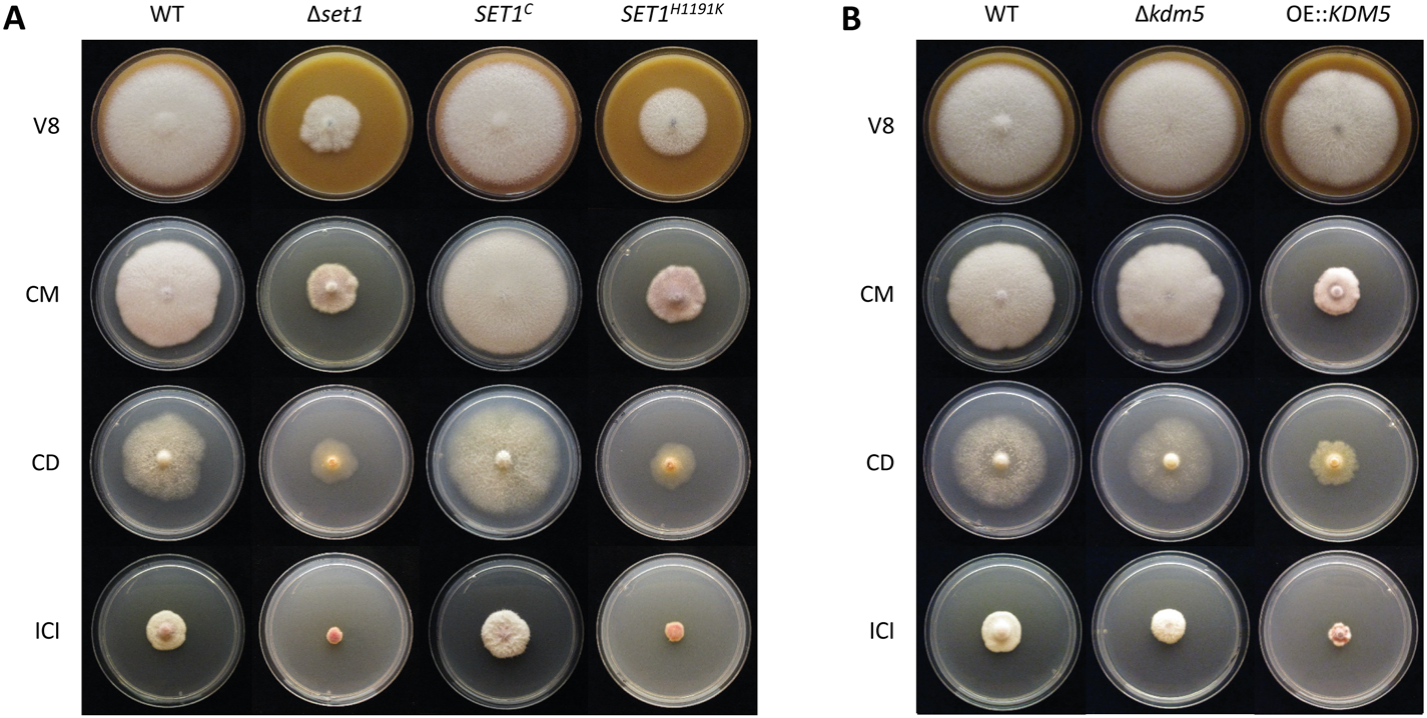
Influence of *SET1* deletion as well as *KDM5* deletion and overexpression on vegetative growth. **A**. The WT, Δ*set1*, *SET1* ^*C*^ and *SET1* ^*H1191K*^ mutants were grown on solid complex (V8, CM) and minimal (CD, ICI + 6 mM NH_4_NO_3_) media for 7 days in the dark in triplicates. **B**. The WT, Δ*kdm5* and OE::*KDM5* mutants were grown under above described conditions.

Next, we performed a plate assay using complex (complete medium, CM and vegetable juice agar, V8) as well as minimal (Czapek Dox, CD and synthetic ICI with 6 mM NH_4_NO_3_) media to assess the impact of H3K4 methylation on vegetative growth. Deletion of *SET1* resulted in a growth defect on all tested media, which was restored in *SET1* ^*C*^ but not in *SET1* ^*H1191K*^ strains (Fig. 2A). Therefore, it is likely that the loss of H3K4 methylation is the sole reason for the poor vegetative growth of Δ*set1*.

While Δ*kdm5* mutants showed a WT‐like colony diameter, OE::*KDM5* mutants exhibited an attenuated growth (Fig. 2B), thereby resembling Δ*set1* and *SET1* ^*H1191K*^. In conclusion, strains with reduced (OE::*KDM5*) or abolished (Δ*set1*, *SET1* ^*H1191K*^) H3K4me3 levels (Fig. 1C) showed retarded hyphal growth, suggesting a correlation between H3K4me3 and vegetative growth in *F. fujikuroi*.

### *Only a small set of genes is antagonistically affected in Δ*set1 *and Δ*kdm5

**Figure 3:**
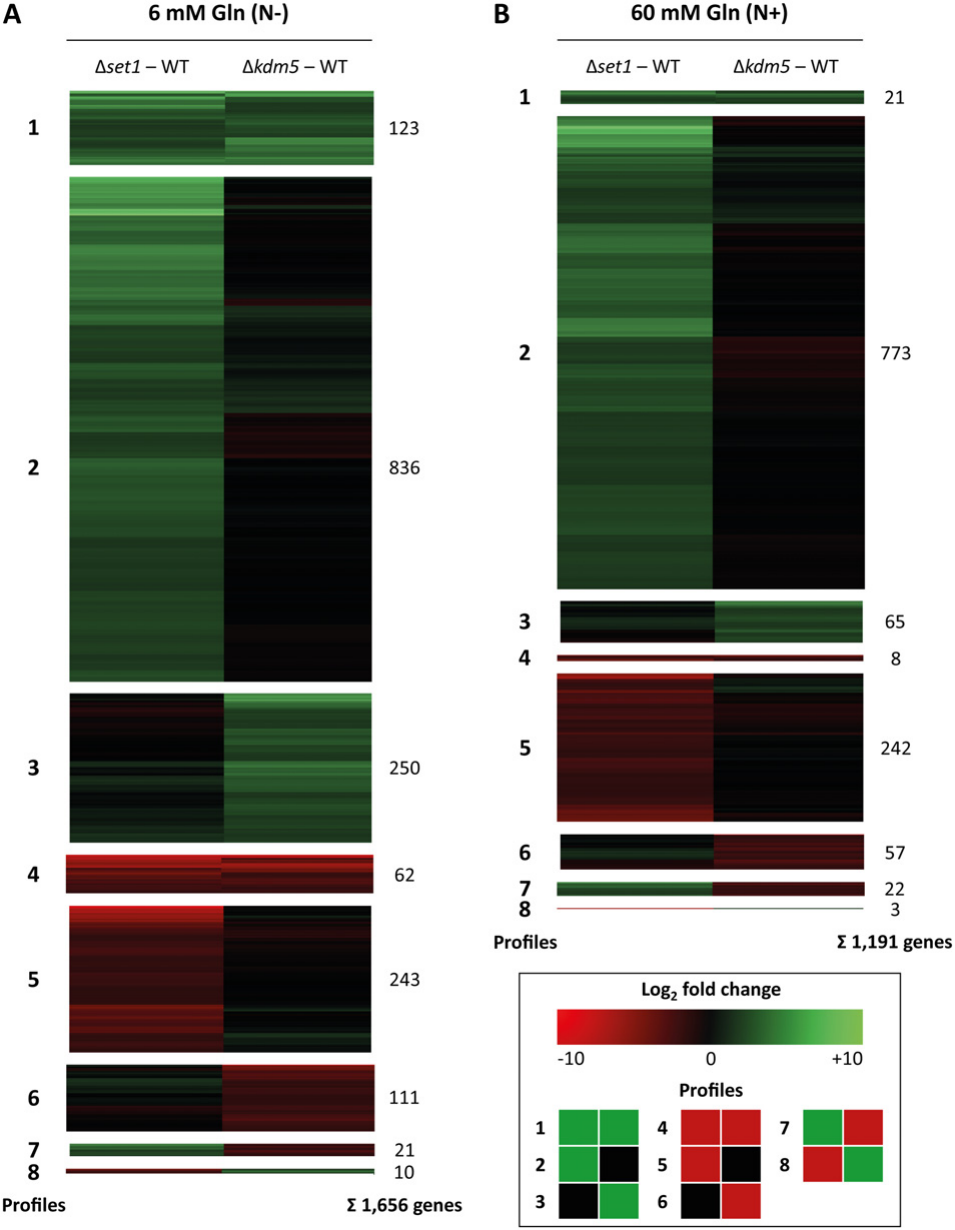
Microarray expression analysis of differentially regulated genes in Δ*set1* and Δ*kdm5*. The WT and the two deletion mutants were grown in ICI liquid culture in the presence of (**A)** limiting (6 mM, N−) and (**B)** saturating (60 mM, N+) amounts of Gln for 3 days prior to RNA extraction. Data are mean values (*n* = 2). Genes upregulated in the deletion mutants compared with the WT are green (log_2_ fold change ≥ 2), downregulated genes are red (log_2_ fold change ≤ −2), and not differentially expressed genes are black (between −2 and 2). The eight profiles were extracted first, and then the genes were clustered for each profile.

In order to compare the genome‐wide impact of *SET1* and *KDM5* deletion on transcription, we performed a microarray expression analysis. For this, the WT, Δ*set1* and Δ*kdm5* mutants were cultivated in synthetic ICI liquid medium with limiting (6 mM, N−) or saturating (60 mM, N+) amounts of glutamine (Gln), conditions that were previously shown to either induce or repress the expression of several SM genes in *F. fujikuroi* (Wiemann *et al*., 2013; Niehaus *et al*., 2017a). Based on the selection criteria of a fourfold change in expression (log_2_ fold change ≥ 2 or ≤ −2) at the 95% confidence interval (false discovery rate *<* 0.05), 2282 of the 14,816 annotated genes (15.4%) were affected in a Set1‐ and/or Kdm5‐dependent manner in at least one condition. Among these, 1,656 and 1,191 genes were differentially expressed (compared with the WT) in the presence of 6 and 60 mM Gln, respectively, giving an overlap of 565 genes affected under both nitrogen conditions (Fig. 3).

The majority of genes was regulated (directly or indirectly) by Set1, while only a smaller set of genes was affected by Kdm5, and an even smaller set of genes was regulated by both Set1 and Kdm5. More precisely, under nitrogen limitation, 1,079 genes (65.2%) were only affected in Δ*set1*, 361 genes (21.8%) were only affected in Δ*kdm5* and 216 genes (13.0%) were up‐ and/or downregulated in both Δ*set1* and Δ*kdm5* (Fig. 3A). Notably, the majority of genes was upregulated upon *SET1* and/or *KDM5* deletion (73.0% in Fig. 3A), whereas fewer genes were downregulated (25.1% in Fig. 3A). In contrast to our expectation, only a small set of genes was regulated in an antagonistic manner by Set1 and Kdm5 (1.9% in Fig. 3A and 2.1% in Fig. 3B). At this point, no direct regulation mechanisms can be concluded from these data, as the observed changes in gene expression can still be mediated by downstream regulators, and not via direct binding to the loci by Set1 and Kdm5.

Under both nitrogen conditions, the largest group comprised genes upregulated in Δ*set1* but not affected in Δ*kdm5*: this profile 2 contained 50.5% and 64.9% of all differentially regulated genes in the presence of 6 and 60 mM Gln respectively (Fig. 3). A Functional Catalogue (FunCat) analysis (Ruepp *et al*., 2004) to identify significantly enriched protein functions in this profile identified the FunCat groups ‘01.20 secondary metabolism’ and ‘01 metabolism’ under both nitrogen conditions (Supporting Information Table S1). Furthermore, in the presence of 6 mM Gln, four additional FunCat groups were enriched with a high significance in profile 2: ‘01.05 C‐compound and carbohydrate metabolism’, ‘01.06.05 fatty acid metabolism’, ‘02.07 pentose‐phosphate pathway’ and ‘32.07 detoxification’ (Supporting Information Table S1).

In summary, the genome‐wide transcriptome analysis yielded interesting insights: the majority of genes is up‐ but not downregulated upon deletion of *SET1*, including several SM‐related genes. Set1 and Kdm5 act only in a minority of cases as true antagonists, either directly or indirectly on a transcriptional level.

### *WT‐like SM biosynthesis depends on both Set1 and Kdm5*

Based on the microarray analysis, several SM key enzyme‐encoding genes are differentially expressed in Δ*set1* and/or Δ*kdm5* in comparison to the WT. Strikingly, 15 out of the 21 affected key genes are responsive to nitrogen in the WT, and are preferentially expressed under either nitrogen limitation or surplus conditions (Supporting Information Table S2). In agreement with the genome‐wide transcriptome analysis (Fig. 3), several SM key genes were found to be upregulated in Δ*set1*. In fact, 8 out of 12 and 5 out of 8 genes were upregulated in the Δ*set1* mutant compared with the WT under N− and N+ conditions respectively (Supporting Information Table S2). Among those are several so far cryptic SM key genes without yet assigned products, that is, *PKS‐NRPS9*, *PKS type III*, *NRPS4*, *NRPS23* and *DMATS3*, rendering this strain an interesting target for future product analyses. Fewer SM genes were affected by the loss of *KDM5*. Here, only 8 SM key genes were deregulated in Δ*kdm5* with the majority of them (5 out of 8) being downregulated (Supporting Information Table S2). Noteworthy, only two of them, that is, *PKS4* and *NRPS31*, involved in the biosynthesis of BIK and APF, respectively, were found to be regulated in an antagonistic manner by Set1 and Kdm5. Indeed, not only the key enzyme‐encoding genes, but all 6 BIK cluster genes and 11 APF cluster genes were similarly affected by *SET1* and *KDM5* deletion (Supporting Information Fig. S2).

**Figure 4:**
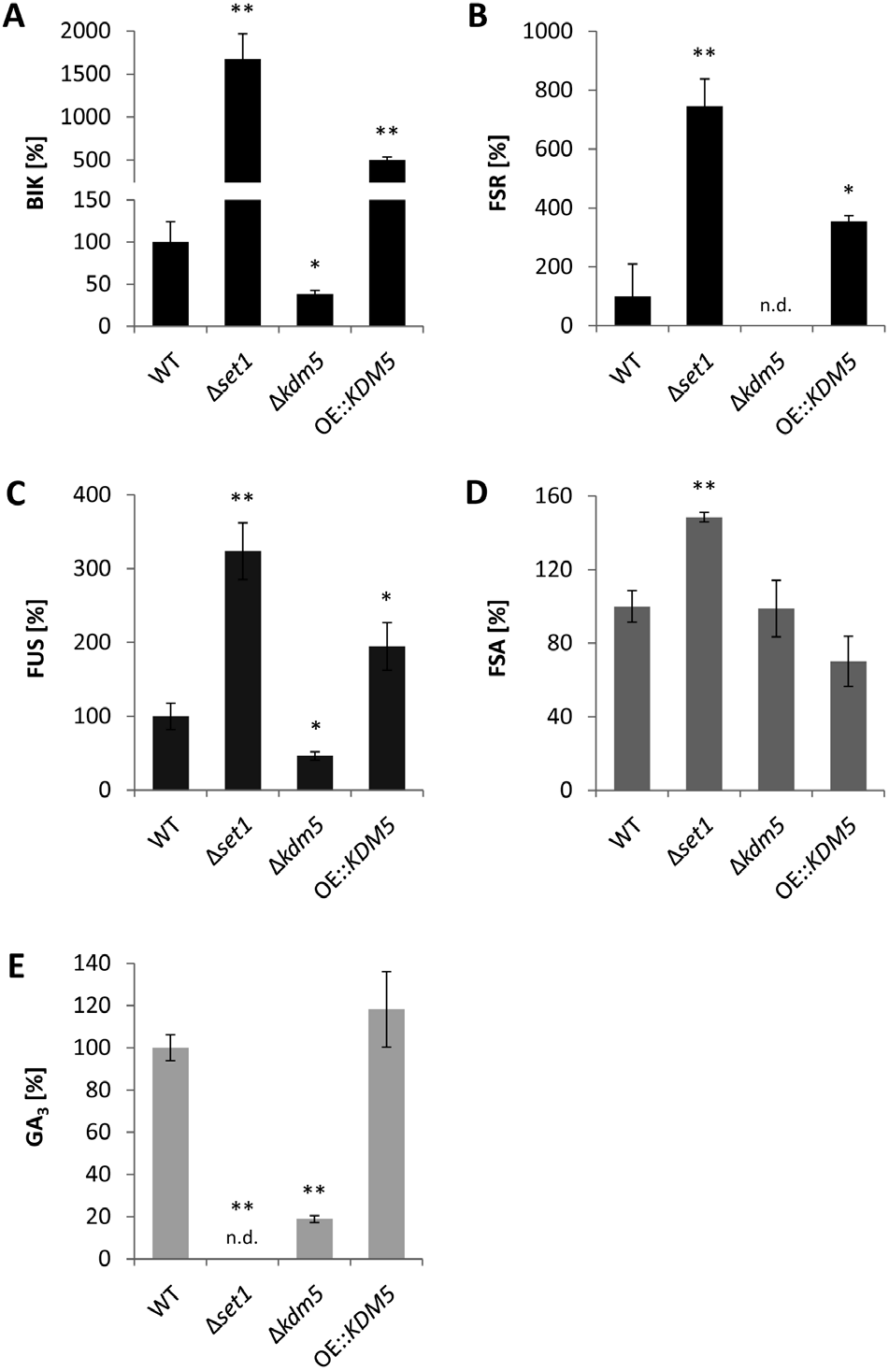
SM biosynthesis is deregulated in Δ*set1*, Δ*kdm5* and OE::*KDM5* mutants. Indicated strains were grown in ICI liquid culture for 7 days and analysed via HPLC‐DAD. The cultivation was done in the presence of 6 mM Gln for bikaverin (BIK) and gibberellic acid GA_3_ (**A, E**), 6 mM NaNO_3_ for fusarubins (FSR) (**B**) or 60 mM Gln for fusarins (FUS) and fusaric acid (FSA) (**C, D**). The production was related to the dry weight of the strains and the production level of the WT was arbitrarily set to 100%. Data are mean values ± SD (*n* = 3). For statistical analysis, the mutants were compared with the WT using the student's *t*‐test: *, *p* < 0.05; **, *p* < 0.01.; n.d., not detected.

Among the known SMs, which can be readily quantified in liquid cultures via high‐performance liquid chromatography coupled to diode array detection (HPLC‐DAD), production of the two red pigments BIK and FSR was shown to be increased in Δ*set1* and OE::*KDM5*, and decreased in Δ*kdm5* compared with the WT under their respective producing conditions (Fig. 4A,B). A similar pattern was observed for the mycotoxin FUS: FUS biosynthesis was downregulated in Δ*kdm5*, and upregulated in the other two strains (Fig. 4C). In contrast, production of the mycotoxin FSA was only slightly enhanced in Δ*set1*, slightly decreased in OE::*KDM5* and not affected in Δ*kdm5* (Fig. 4D). Notably, the biosynthesis of the bioactive phytohormone gibberellic acid GA_3_ showed a different pattern: GA_3_ was not detectable in Δ*set1*, its levels were reduced to about 20% in Δ*kdm5*, and not affected in the *KDM5* overexpression strain (Fig. 4E). Therefore, Set1 and Kdm5 were found to antagonize BIK, FSR, FUS (via HPLC) and possibly APF biosynthesis (according to the microarray). Not all of the described effects on SM production were reflected as significant changes in the microarray expression analysis: the GA key gene was only downregulated in Δ*set1* but not in Δ*kdm5* (6 mM Gln), and the changes for the FUS key gene were not significant (60 mM Gln; Supporting Information Table S2). Furthermore, the producing condition for FSR (6 mM NaNO_3_) was not included in the microarray. The discrepancy between SM gene expression and SM production levels can likely be explained by the fact that the two analyses were performed at different time points, that is, after 3 and 7 days of cultivation respectively. While production levels show the accumulation of a metabolite within an extended time period, gene expression levels stand for one (representative) time point.

BIK, FSR, FSA and GA clusters are located within, and the FUS cluster is located in close proximity to subtelomeric regions of facultative heterochromatin, indicated by the heterochromatic mark H3K27me3 (Supporting Information Fig. S3; Studt *et al*., 2016b). Previous genome‐wide ChIP‐sequencing analysis revealed the absence of H3K4me2 at most of the here analyzed SM genes, except for the GA gene cluster. Here, two out of the seven GA cluster genes were shown to be decorated with H3K4me2 in the WT background only under inducing conditions (Supporting Information Fig. S3C; Wiemann *et al*., 2013). To gain a deeper insight into the H3K4 methylation pattern, we performed ChIP with subsequent quantitative real‐time polymerase chain reaction (qPCR) to determine H3K4me3 and H3K4me2 levels at GA cluster genes in Δ*set1*, Δ*kdm5* and OE::*KDM5* mutants under GA‐inducing conditions. The key gene *CPS/KS* as well as *P450‐1* were chosen as highly expressed GA cluster genes, while *P450‐2* and *P450‐4* were the ones decorated with H3K4me2 under inducing conditions (Supporting Information Fig. S3C).

**Figure 5:**
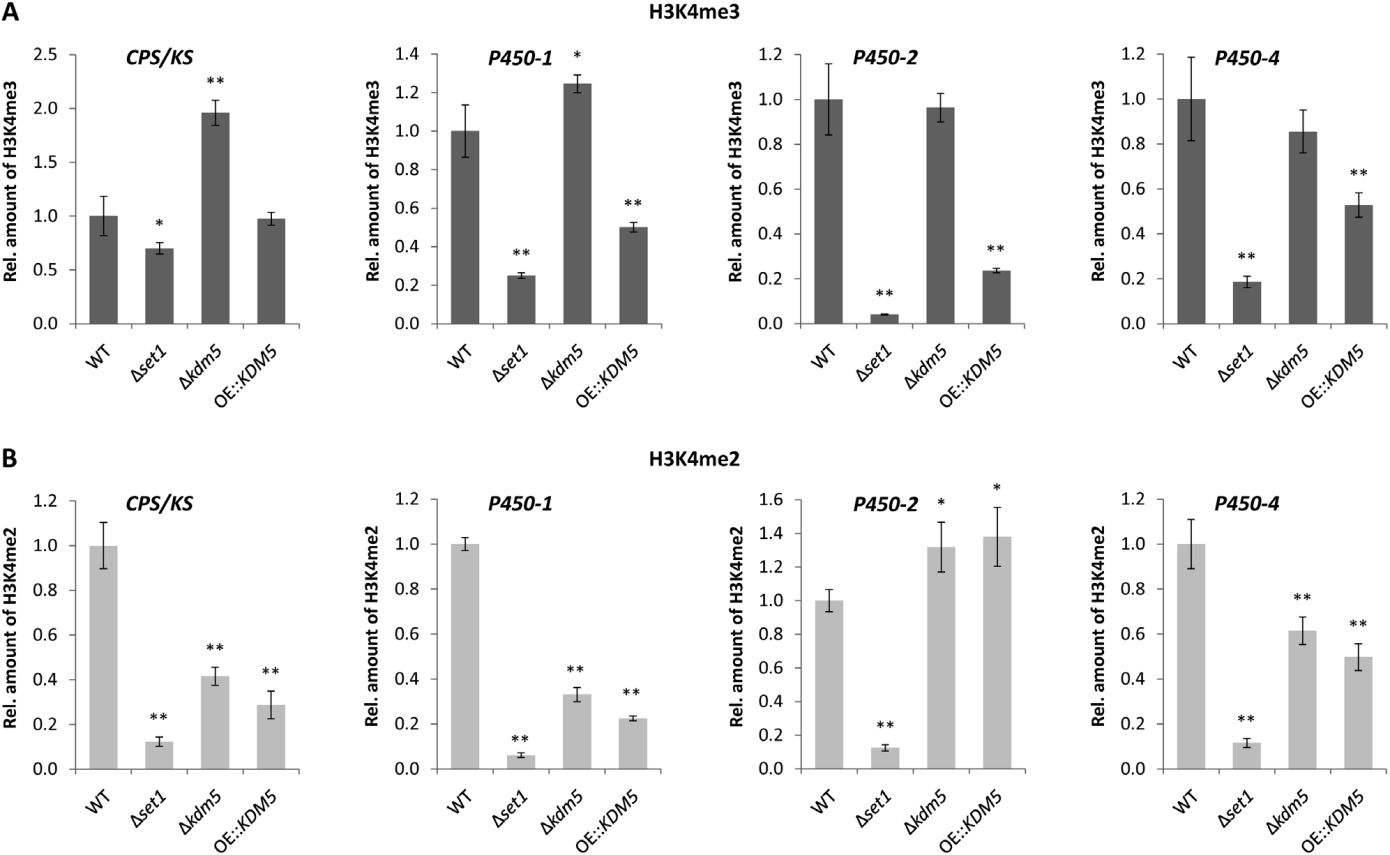
H3K4me3 and H3K4me2 levels at gibberellic acid (GA) cluster genes in Δ*set1*, Δ*kdm5* and OE::*KDM5* mutants. Indicated strains were grown in liquid culture (ICI + 6 mM Gln) for 3 days prior to ChIP‐qPCR using the (**A**) H3K4me3 or (**B**) H3K4me2 antibody. Therefore, the enriched samples (precipitated by antibody) were normalized to the respective input samples (initially applied chromatin). The WT was arbitrarily set to 1, and the data are mean values ± SD (*n* = 4). For statistical analysis, the mutants were compared with the WT using the student's *t*‐test: *, *p* < 0.05; **, *p* < 0.01.

As expected, lack of *SET1* resulted in an overall decrease of H3K4me3 and H3K4me2 at all analysed GA genes (Fig. 5A,B), a phenotype that goes in line with the global loss of H3K4me3/me2 as shown by western blot (Fig. 1C). While H3K4me3 levels followed a similar pattern in Δ*set1* and OE::*KDM5*, and were reduced in the ChIP and western blot analyses (Figs. 1C and 5A), a significant increase in H3K4me3 was only observed for *CPS/KS* and *P450‐1*, but not *P450‐2* and *P450‐4*, in Δ*kdm5* (Fig. 5A). This might be due to relatively high amounts of H3K4me3 at these two genes in the WT background, which cannot be elevated any further in Δ*kdm5*.

Contrary to H3K4me3, H3K4me2 levels were comparable in Δ*kdm5* and OE::*KDM5* at GA cluster genes (Fig. 5B), while both H3K4 methylation marks behaved antagonistically genome‐wide as shown by western blot (Fig. 1C). The effects were more obvious for the GA cluster genes *P450‐2* and *P450‐4*, which exhibit a strong accumulation of H3K4me2 in the WT background (Supporting Information Fig. S3C), and followed a similar trend for *CPS/KS* and *P450‐1* (Fig. 5B). Bearing in mind that Δ*kdm5* and OE::*KDM5* showed a similar phenotype and both produced significant amounts of GA_3_ in axenic culture (Fig. 4C), this might rely on comparable levels of H3K4me2, but not H3K4me3, in these two strains (Fig. 5A,B).

To conclude, secondary metabolism in *F. fujikuroi* was found to depend on functional Set1 and Kdm5: the biosynthesis of several SMs was deregulated in the analyzed mutants Δ*set1*, Δ*kdm5* and OE::*KDM5*. However, the exact regulatory circuits remain to be elucidated.

### *Deletion of* SET1 *results in an attenuated virulence on rice*

**Figure 6:**
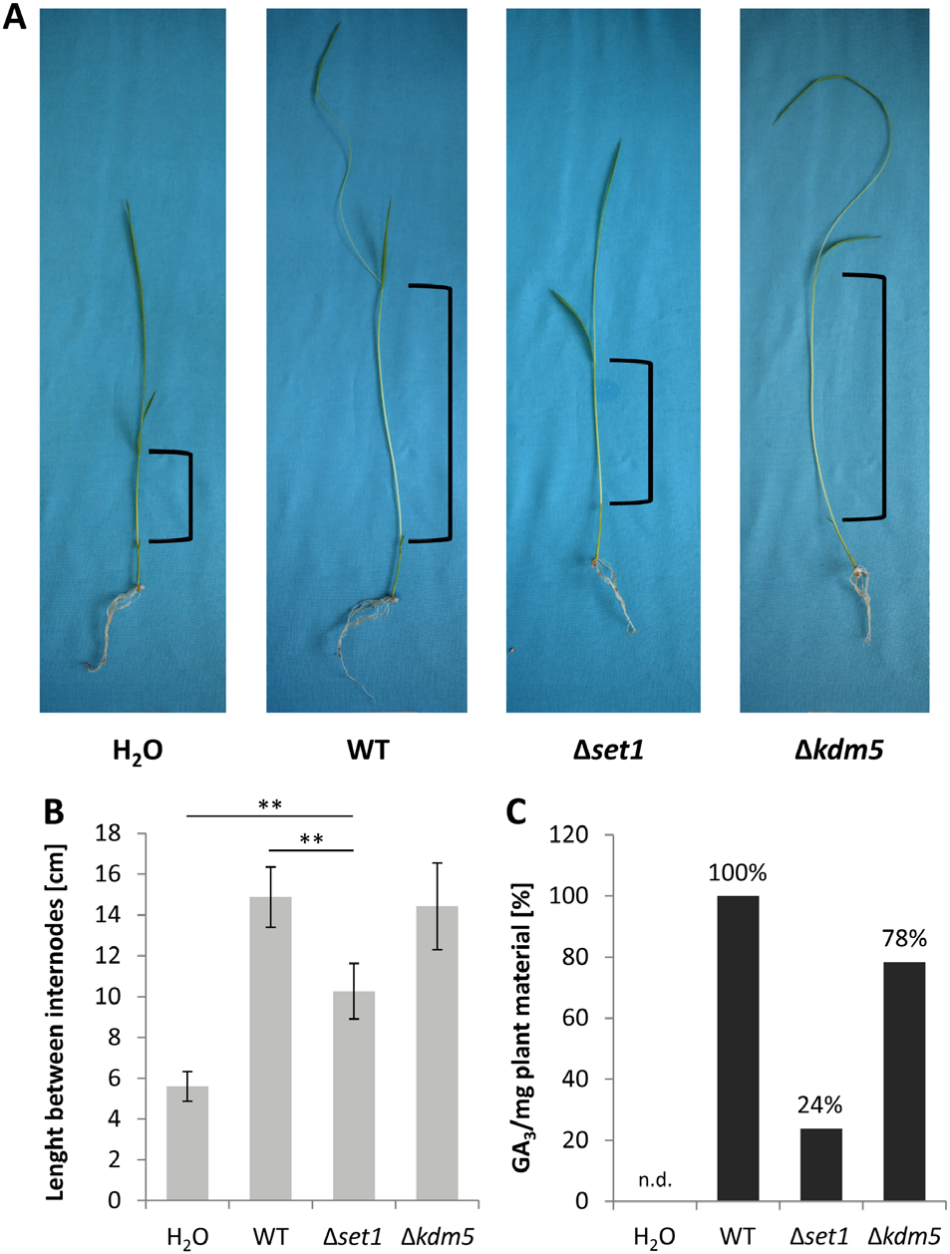
Determination of virulence and GA_3_ levels of Δ*set1*‐ and Δ*kdm5*‐infected rice plants. **A**. Germinated rice seedlings were infected with the WT, the Δ*set1* and Δ*kdm5* strains. Mock infection was achieved through the addition of H_2_O and served as negative control. Progression of infection was determined after 7 days. **B**. The length between rice internodes was measured (indicated in A). Data are mean values ± SD (*n* = 5). For statistical analysis, Δ*set1* was compared with the WT and mock control using the student's *t*‐test: **, *p* < 0.01. **C**. Gibberellic acid GA_3_ levels *in planta* were quantified via HPLC‐MS/MS and normalized to the input plant material. The 5 (infected) rice plants were combined, and then divided again to yield a technical replicate for the SM extraction and quantification. The WT was arbitrarily set to 100%. n.d., not detected.

Most *F. fujikuroi* strains cause *bakanae* disease when infecting rice plants as a result of their secretion of GAs, a group of highly bioactive phytohormones (Niehaus *et al*., 2017a). Since the GA_3_ production in axenic culture was fully abolished in Δ*set1* and significantly decreased in Δ*kdm5* (Fig. 4E), we next performed infection studies of the analysed mutant strains. Therefore, surface‐sterilized and pre‐germinated rice seedlings were infected with the WT, Δ*set1*, Δ*kdm5* and OE::*KDM5* strains (Fig. 6; Supporting Information Fig. S4). Both Δ*kdm5* and OE::*KDM5* resulted in a WT‐like phenotype, characterized by chlorotic and hyper‐elongated rice internodes (Fig. 6A,B; Supporting Information Fig. S4), albeit the significantly reduced GA_3_ levels found in Δ*kdm5* strains during axenic growth (Fig. 4E). Notably, even the rice plants infected with Δ*set1*, a strain that did not produce detectable GA_3_ levels during axenic growth (Fig. 4E), showed slightly chlorotic and extended internodes (Fig. 6A,B). Subsequent chemical quantification of GA_3_ levels *in planta* revealed that GA_3_ levels were in fact not abolished in Δ*set1* during the infection. Rice samples infected with Δ*set1* contained 24% of the GA_3_ level of WT‐infected rice samples (Fig. 6C). Notably, GA_3_ levels in Δ*kdm5*‐infected plant material were also higher compared with axenic growth (78% of WT‐infected samples instead of 20% in axenic culture) (Figs. 4E and 6C), thereby likely explaining the WT‐like infection pattern of this strain (Fig. 6A,B).

### *Set1 and Kdm5 are positive and negative regulators of conidiation, respectively*

The formation of asexual spores is an important way for filamentous, pathogenic fungi to proliferate and spread. The *F. fujikuroi* WT IMI58289 used in this work produces only microconidia (here simply referred to as ‘conidia’), while other strains produce micro‐ and macroconidia (with several septa), or predominantly macroconidia (Niehaus *et al*., 2017a). As conidiation‐related genes are part of the conserved fungal genome, we assumed an effect on conidiogenesis upon perturbation of the euchromatic H3K4 methylation mark in *SET1* and *KDM5* mutants.

**Figure 7:**
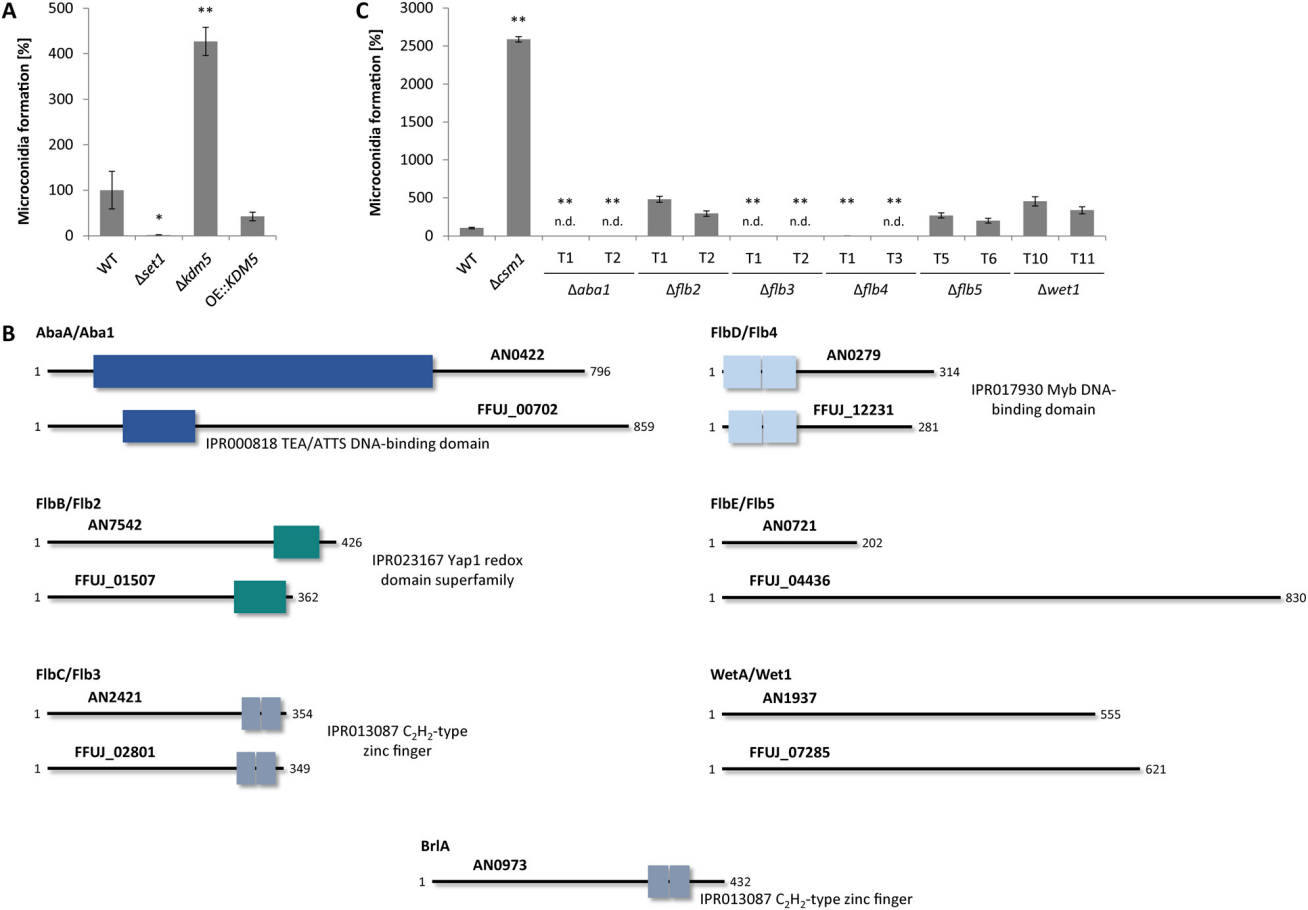
Conidiation is affected by *SET1* and *KDM5* deletion, and is regulated by the conserved TFs Aba1, Flb3 and Flb4. **A**. Indicated strains were grown on V8 agar in the presence of a 12 h light/12 h dark cycle for 14 days prior to analysis of conidia formation. Data are mean values ± SD (*n* = 3). For statistical analysis, the mutants were compared with the WT using the student's *t*‐test: *, *p* < 0.05; **, *p* < 0.01. **B**. The *A. nidulans* regulators AbaA, FlbB‐FlbE and WetA, but not BrlA, are conserved in *F. fujikuroi*. The domain description includes the respective InterPro accession numbers. **C**. Indicated strains were grown and analysed as described above. n.d., not detected.

To assess the ability of Δ*set1*, Δ*kdm5* and OE::*KDM5* mutants to reproduce asexually, we next quantified conidia formation. While both Δ*set1* and OE::*KDM5* produced only very few conidia, Δ*kdm5* showed elevated conidia formation in comparison to the WT, suggesting that Set1 and Kdm5 indeed antagonize conidiogenesis (Fig. 7A).

Intrigued by these findings, we searched the *F. fujikuroi* genome for conidiation‐related genes, which have already been well‐characterized in *A. nidulans* (Krijgsheld *et al*., 2013). The upstream cascade is mainly transmitted by the so‐called ‘fluffy’ proteins, many of which are TFs, such as FlbB (basic leucine zipper TF), FlbC (C_2_H_2_ TF) and FlbD (cMyb TF), while FlbE does not harbor a conserved domain (Fig. 7B). Both FlbC and a heterodimer of FlbB/FlbD (FlbD is released from a heterodimer with FlbE) are required for efficient *brlA* (*bristle*) expression, which encodes the central regulator of asexual development in *A. nidulans*. BrlA further activates the TF gene *abaA* (*abacus*), which in turn activates *wetA* (*wet white*), the two being required for conidia formation and maturation respectively (Krijgsheld *et al*., 2013).

Orthologs for *A. nidulans flbB*‐*flbD* were determined by QuartetS (Yu *et al*., 2011), while no entry was found for *abaA*, *flbE* and *wetA*. Thus, here putative orthologs were identified as ‘Best‐Hits’ by BlastP analyses. No putative ortholog for *brlA* could be identified in the genome of *F. fujikuroi* (Fig. 7B). BrlA seems to be absent from the genus *Fusarium* (Son *et al*., 2014a; Niehaus *et al*., 2017a). Single deletion mutants were generated for the *F. fujikuroi* orthologs, designated *ABA1*, *FLB2*‐*FLB5* and *WET1*, and the mutants were tested for their ability to produce conidia. In Fig. 7C, the novel mutants are shown in comparison to Δ*csm1* (regulator of *c*onidiation and *s*econdary *m*etabolism). The TF‐encoding gene *CSM1* encodes a major repressor of conidiation in *F. fujikuroi*, and the respective deletion strain is characterized by a strongly elevated conidia formation relative to the WT (Niehaus *et al*., 2017b). Indeed, conidiogenesis was abolished in Δ*aba1*, Δ*flb3* and Δ*flb4* mutants, while WT‐like amounts of conidia were produced in Δ*flb2*, Δ*flb5* and Δ*wet1* (Fig. 7C). A plate assay performed under conidiation‐inducing conditions did not show an altered phenotype, concerning the colony diameter or morphology, for any of the generated deletion mutants (Supporting Information Fig. S5A). Furthermore, a microscopic analysis revealed that Wet1 is probably also involved in conidia separation and maturation in *F. fujikuroi*, as Δ*wet1* mutants did not form longer strings of conidia as the WT, but formed smaller nests of conidia that remained close to the vegetative hyphae (Supporting Information Fig. S5B).

As indicated above, *F. fujikuroi* IMI58289 analyzed so far produces only microconidia, while the sequenced strain *F. fujikuroi* E282 produces macroconidia (Niehaus *et al*., 2017a). To elucidate whether Aba1 might be also required for macroconidia formation in E282, the respective *ABA1* ortholog *FFE2_00769* was deleted also in the E282 background. Indeed, macroconidia formation was fully abolished in E282/Δ*aba1* mutants (data not shown), suggesting that the synthesis of micro‐ and macroconidia underlies similar transcriptional regulation mechanisms in *Fusarium* spp.

**Figure 8:**
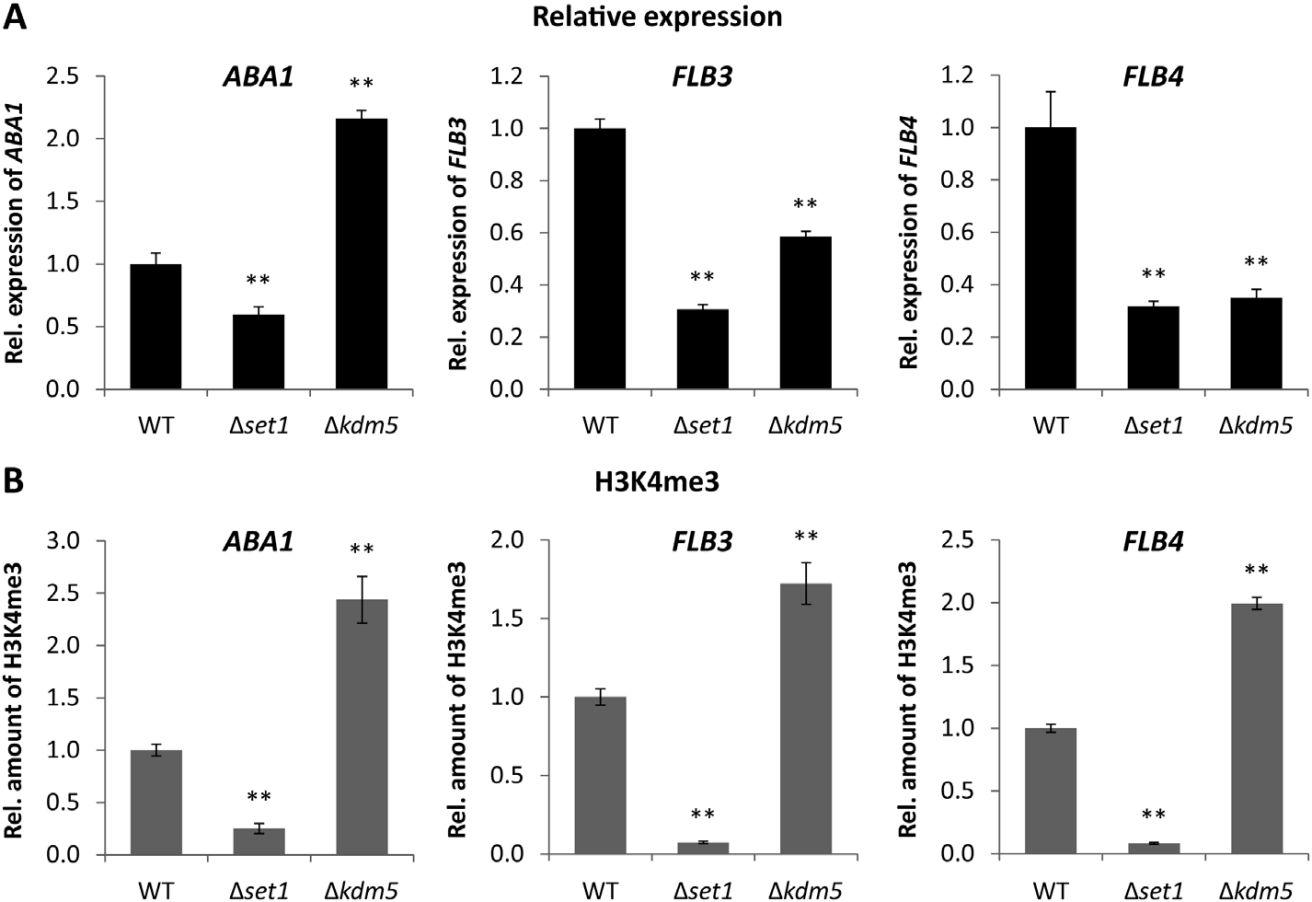
Relative expression as well as H3K4me3 levels of conidiation‐related TF genes in Δ*set1* and Δ*kdm5* mutants. Indicated strains were grown on V8 agar in the presence of a 12 h light/12 h dark cycle for 3 days to induce conidiation. **A**. RNA was extracted and the relative expression of *ABA1*, *FLB3* and *FLB4* was determined by RT‐qPCR. **B**. ChIP‐qPCR was performed for the same genes using the H3K4me3 antibody. Therefore, the enriched samples (precipitated by antibody) were normalized to the respective input samples (initially applied chromatin). The WT was arbitrarily set to 1, and the data are mean values ± SD (*n* = 4). For statistical analysis, the mutants were compared with the WT using the student's *t*‐test: *, *p* < 0.05; **, *p* < 0.01.

To elucidate whether Set1 and Kdm5 regulate any of the identified conidiation‐specific TF‐encoding genes, *ABA1*, *FLB3* or *FLB4*, the relative expression of these three genes was assessed in Δ*set1* and Δ*kdm5* mutants in comparison to the respective WT IMI58289 under conidiation‐inducing light conditions. Moreover, ChIP‐qPCR was performed to identify changes in H3K4me3 at these genes, which are located in conserved euchromatic regions of chromosome I (*ABA1*), chromosome III (*FLB3*) and chromosome VIII (*FLB4*). Only the expression of *ABA1*, but not of *FLB3* or *FLB4*, fitted to the conidia production levels of the mutants at the time point chosen: *ABA1* was downregulated in Δ*set1* and upregulated in Δ*kdm5* after 3 days of incubation (Fig. 8A), correlating with decreased and increased conidia formation in these mutants respectively (Fig. 7A). Indeed, the deposition of H3K4me3 correlated with the detected *ABA1* expression and conidia production levels of the mutants (Fig. 8B). The distribution of H3K4me3 at the genes *FLB3* and *FLB4* showed the same trend (Fig. 8B), and it cannot be excluded that their expression exhibited the same profile at an earlier time point, with H3K4me3 indicating recently performed gene expression.

To summarize, Set1 and Kdm5 were identified as activator and repressor of conidiation, respectively, which likely regulate *ABA1* expression via deposition of H3K4me3. In addition to Aba1, Flb3 and Flb4 act as positive TFs in conidia formation, while Wet1 is probably involved in conidia maturation.

### Fusarium fujikuroi ABA1 *is the major conidiation‐specific TF gene targeted by global regulators*

**Figure 9:**
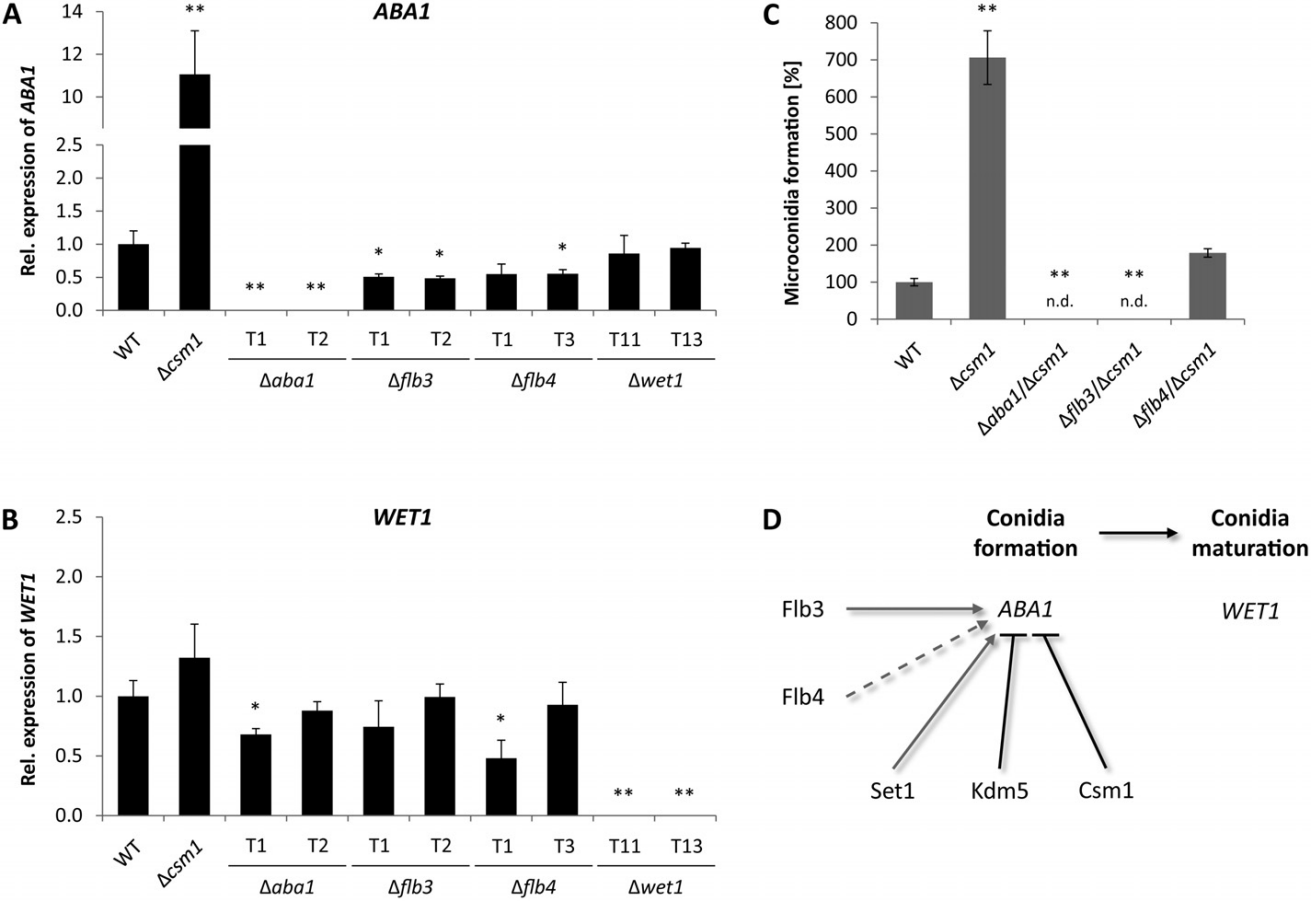
Regulatory network of conidiation. **A,B**. The strains were grown on V8 agar in the presence of a 12 h light/12 h dark cycle for 3 days. Then, RNA was extracted and the relative expression of *ABA1* and *WET1* was determined by RT‐qPCR. Data are mean values (*n* = 3). **C**. Indicated strains were grown for 14 days under above described conditions prior to analysis of conidia formation. Data are mean values ± SD (*n* = 4). For statistical analysis, the mutants were compared with the WT using the student's *t*‐test: *, *p* < 0.05; **, *p* < 0.01; n.d., not detected. **D**. Current model of conidia formation and maturation in *F. fujikuroi*, with *ABA1* as major target of positive and negative regulators of conidiation. Dashed lines indicate that Flb4 can be overruled by Csm1.

In order to gain a deeper insight into the hierarchical network of regulation, we performed a qPCR expression analysis of Δ*aba1*, Δ*flb3*, Δ*flb4* and Δ*wet1*, in comparison to the WT and Δ*csm1*, grown under conidiation‐inducing conditions. The analysis revealed that *ABA1* is likely the major target of these regulators: its expression is induced by Flb3 and Flb4 (downregulated in the deletion mutants), whereas it is strongly repressed by Csm1 in the WT background (upregulated in Δ*csm1*) (Fig. 9A). Furthermore, *WET1* was neither significantly deregulated in Δ*aba1* nor in any other mutant strain at the time point chosen (Fig. 9B).

Earlier investigations have revealed several global regulators with an influence on asexual development in *F. fujikuroi*. Thus, the fungus‐specific *velvet* protein Vel1 and the GATA‐type TF Csm1 were shown to be an activator and repressor of conidiation respectively (Wiemann *et al*., 2010; Niehaus *et al*., 2017b) (Fig. 9A). *CSM1* deletion in a Δ*vel1* background (no conidia) restored conidiogenesis (Niehaus *et al*., 2017b). To analyze if lack of the repressor gene *CSM1* also restores conidiogenesis in Δ*aba1*, Δ*flb3* and Δ*flb4*, *CSM1* was deleted in the respective mutant strains in the present work. Both Aba1 and Flb3, but not Flb4, were strictly required for asexual development, and the loss of conidia formation in these mutants was not overcome by deleting *CSM1* (Fig. 9C).

Based on these data, we have established the following working model that is depicted in Fig. 9D: Flb3 is strictly required, but also Flb4 is a positive regulator of *ABA1* expression. In contrast, Csm1 represses the expression of *ABA1*, which is responsible for conidia formation. Subsequently, Wet1 induces conidia maturation. Furthermore, we have identified two novel regulators of conidiation: Set1 as activator and Kdm5 as repressor of *ABA1* expression.

## Discussion

In the present work, we analyzed *F. fujikuroi* Set1, the methyltransferase of the H3K4‐specific COMPASS complex, which was shown to be responsible for all detectable H3K4me2/me3 genome‐wide (and for the majority, if not all, of H3K4me1). As a counterpart, we generated single deletion and overexpression mutants of *F. fujikuroi KDM5*, encoding the ortholog to *S. cerevisiae* Jhd2 and *A. nidulans* KdmB (Liang *et al*., 2007; Gacek‐Matthews *et al*., 2016). Similarly to Jhd2 and KdmB, Kdm5 was shown to remove H3K4me3 *in vivo*, resulting in a genome‐wide increase and decrease of H3K4me3 in Δ*kdm5* and OE::*KDM5* mutants respectively. The remaining H3K4me2/me1 marks are likely to be demethylated by an Lsd1‐type amine oxidase in *F. fujikuroi*, which was first described for *Schizosaccharomyces pombe* (Shi *et al*., 2004).

### *Set1 and Kdm5 affect genome‐wide transcription and vegetative growth*

A transcriptome analysis of Δ*set1* and Δ*kdm5* mutants in comparison to the *F. fujikuroi* WT revealed that the two enzymes act as true antagonists only in the minority of cases, using the strong selection criterion of a fourfold change in expression. However, we did find more subtle antagonistic roles of Set1 and Kdm5 in SM biosynthesis and conidiation, as further discussed below. In the genome‐wide expression analysis, the majority of affected genes was upregulated upon both *SET1* and *KDM5* deletion. This was surprising regarding the fact that the euchromatic, activating mark H3K4me3 was antagonistically affected in western blot analyses, being decreased in Δ*set1* and increased in Δ*kdm5* mutants respectively. Of course, many of the observed effects on gene expression are likely to be secondary, and to be mediated by unknown downstream regulators, such as Set1‐dependent repressors in this case. Similarly, genome‐wide transcription was both up‐ and downregulated upon deletion of *F. fujikuroi GCN5* that is responsible for the majority of the activating acetylation (ac) marks, for example, H3K9ac and H3K27ac (Rösler *et al*., 2016). This was also true for the deletion of the two histone deacetylase (HDAC) genes *HDA1* and *HDA2* in this fungus (Studt *et al*., 2013).

In the performed microarray, the largest group was profile 2 under both nitrogen quantities tested: genes upregulated in Δ*set1* and not differentially expressed in Δ*kdm5* (compared with the WT in each case). Several FunCat groups were identified to be significantly enriched in this profile, including genes of primary and secondary metabolism as well as of detoxification pathways. Also, a significant number of cryptic SM key genes was shown to be upregulated in Δ*set1*. Thus, either the perturbation of carbohydrate and fatty acid metabolism, or the upregulation of possibly toxic SMs could be the reason for the slow growth of Δ*set1* mutants in plate assays. Something similar was suggested for the crippled *F. graminearum* Δ*kmt6* mutant that exhibited a downregulation of the repressive mark H3K27me3 and as a consequence, an upregulation of cryptic SM gene clusters (Connolly *et al*., 2013). In the performed microarray, *PKS‐NRPS1*, encoding the key enzyme in trichosetin biosynthesis, was upregulated in Δ*set1* under nitrogen limitation. The tetramic acid trichosetin is a potent mycotoxin, which was shown to inhibit vegetative growth when overproduced by *F. fujikuroi* (Janevska *et al*., 2017). Noteworthy, *PKS‐NRPS1* expression was similarly induced in *F. fujikuroi KMT6* knock‐down mutants, also exhibiting poor vegetative growth (Studt *et al*., 2016b).

The *in loco* complementation of Δ*set1* with the point‐mutated variant *SET1* ^*H1191K*^ did not restore H3K4 methylation levels and notably, the observed growth defect, suggesting that the two are correlated. OE::*KDM5* mutants were also characterized by a slow growth, indicating that the depletion of H3K4me3 in Δ*set1*, *SET1* ^*H1191K*^ and OE::*KDM5* mutants could be associated with this phenotype. A slight reduction in colony diameter was also observed for Δ*ccl1* in both *F. fujikuroi* and *F. graminearum* (Studt *et al*., 2017), further strengthening the assumption that reduced H3K4me3 levels are responsible for growth retardation. Whether the strong phenotype of OE::*KDM5* might also be correlated with an overexpression of *PKS‐NRPS1*, and therefore partially be attributed to trichosetin overproduction, remains elusive at this point. Noteworthy, colony diameters of *F. graminearum* and *F. verticillioides* Δ*set1* mutants were also reduced in comparison to the respective WT strains (Liu *et al*., 2015; Gu *et al*., 2017).

### *Set1 and Kdm5 regulate SM biosynthesis mostly in an indirect manner*

As indicated above, secondary metabolism was strongly affected upon *SET1* deletion, but also upon *KDM5* deletion and overexpression. The strength of the present study is that we were able to compare the different and possibly antagonizing phenotypes with each other. Notably, production of the two red pigments BIK and FSR as well as of the mycotoxin FUS was regulated in an antagonistic manner by Set1 and Kdm5: all three SMs were upregulated in Δ*set1* and OE::*KDM5*, but downregulated in Δ*kdm5* in comparison to the WT. This suggests a repressive influence of H3K4me3 on the respective SM gene clusters in the WT background, because this mark was decreased in both Δ*set1* and OE::*KDM5*, and accordingly increased in Δ*kdm5* genome‐wide. These results are in accordance with a previous study, where we have observed an increase in BIK and FUS biosynthesis (FSR was not tested) upon deletion of *CCL1*, also showing reduced levels of H3K4me3 genome‐wide (Studt *et al*., 2017). However, in Δ*ccl1*, H3K4me2/me3 levels were not affected at FUS cluster genes, and H3K4me2 was elevated at BIK cluster genes with unchanged H3K4me3 levels (Studt *et al*., 2017). Contrary to this, the Δ*set1* mutant showed a complete loss of all detectable H3K4me2 and H3K4me3. Therefore, the alterations in H3K4me2 at the BIK genes observed in Δ*ccl1* cannot be the reason for the comparable BIK phenotype in Δ*set1* and Δ*ccl1* mutants. Overall this suggests that the observed phenotypes concerning BIK, FSR and FUS biosynthesis are indeed mediated by the globally changing H3K4me3 levels, however, by an as yet unknown regulatory circuit.

Generally, H3K4 methylation is found within euchromatic regions and is absent from the majority of subtelomeric SM gene clusters in the *F. fujikuroi* WT, also under inducing conditions (Wiemann *et al*., 2013). Something very similar was described also for *F. graminearum* and *A. nidulans* (Connolly *et al*., 2013; Gacek‐Matthews *et al*., 2016), suggesting that most SM gene clusters are rather regulated in an indirect manner, and are not targeted by Set1 or Kdm5, in *F. fujikuroi* and likely also in other fungi. Early work on Set1 and H3K4 methylation in *S. cerevisiae* has revealed a HDAC‐independent silencing mechanism of rDNA, mating type and subtelomeric loci (Briggs *et al*., 2001; Krogan *et al*., 2002; Bryk *et al*., 2002). However, the exact mechanism is still not well understood, and recruitment of the HDAC Sir2 to subtelomeric loci and subsequent hypoacetylation is generally accepted as main silencing mechanism in budding yeast (Erlendson *et al*., 2017). As *Fusarium* species employ H3K9 and especially H3K27 methylation to silence vast stretches of constitutive and facultative heterochromatin, respectively (Connolly *et al*., 2013; Wiemann *et al*., 2013; Studt *et al*., 2016b), it is questionable whether H3K4 methylation further contributes as a silencing mechanism in *F. fujikuroi*. The fact that H3K4 methylation is predominantly absent from facultative heterochromatin at subtelomeres in general, and from silenced SM genes in particular (Wiemann *et al*., 2013), would argue against this hypothesis.

The only subtelomeric SM gene cluster analysed here that harbors H3K4me2 under inducing conditions is the GA gene cluster. Here, two out of the seven cluster genes, *P450‐2* and *P450‐4*, are decorated with H3K4me2 under inducing but not under repressing conditions (Wiemann *et al*., 2013). Indeed, ChIP‐qPCR analyses revealed that these genes are decorated with H3K4me2 in the WT, Δ*kdm5* and OE::*KDM5* mutants, possibly correlating with GA biosynthesis in these strains, while the distribution of H3K4me3 did not fit the production levels.

Accordingly, loss of H3K4me2 in Δ*set1* was accompanied with an abolished GA_3_ production *in vitro*, which correlated with a reduced virulence on rice. Similarly, Set1 was required in both *F. graminearum* and *F. verticillioides* for full virulence on wheat and maize respectively (Liu *et al*., 2015; Gu *et al*., 2017). In *F. graminearum*, the SM deoxynivalenol (DON) is the virulence factor essential for successful progression of wheat infection. While H3K4me3 was mostly absent from DON cluster genes, H3K4me2 levels correlated well with DON gene expression and product formation in the *F. graminearum* WT (producer) and *SET1* mutant (non‐producer) under axenic conditions (Liu *et al*., 2015). Therefore, in both *F. fujikuroi* and *F. graminearum*, H3K4me2 might be important for GA and DON biosynthesis respectively.

While Set1 was required for full virulence of *F. fujikuroi* on its preferred host plant rice, Δ*kdm5*‐infected rice seedling resembled WT‐infected samples. It is noteworthy that Δ*set1* strains still showed *bakanae* symptoms on infected rice samples, though strongly attenuated. This contradictory phenotype can be explained by the finding that although abolished during axenic growth, residual GA_3_ levels of about 20% were detectable in Δ*set1*‐infected rice samples. For Δ*kdm5*, GA_3_ levels were reduced to about 20% in axenic culture, but elevated to about 80% during *in planta* growth, likely explaining the WT‐like virulence of this strain. Similar phenotypes have also been observed for Δ*ccl1* (Studt *et al*., 2017) and Δ*sge1* (Michielse *et al*., 2015) mutants before, with *SGE1* encoding a global SM regulator in *F. fujikuroi*. This induction of GA_3_ biosynthesis *in planta* may be mediated by a specific, possibly plant‐derived signal, thereby suggesting a more complex regulation during the infection process as opposed to axenic growth.

In summary, a significant influence of H3K4 methylation on SM biosynthesis was observed, however, further research is required to determine the mode of action of COMPASS and Kdm5, and how exactly subtelomeric SM gene clusters are affected by these histone modifiers.

### *Set1 and Kdm5 regulate the conidiation‐specific TF gene* ABA1

One of the most intriguing phenotypes of the Δ*set1* and Δ*kdm5* mutants is the abolished and enhanced conidiation respectively. In the present work, we identified the orthologues to the *A. nidulans* conidiation‐related genes *abaA*, *flbB*‐*flbE* and *wetA*, which were designated *F. fujikuroi ABA1*, *FLB2*‐*FLB5* and *WET1*. We showed that Aba1, Flb3 and Flb4 are essential for conidia formation, while Flb2 and Flb5 are dispensable in *F. fujikuroi*. In *A. nidulans*, the Δ*flbB*‐Δ*flbE* mutants were characterized by a fluffy phenotype and a low *brlA* expression, and therefore an abolished or delayed conidiogenesis (Wieser *et al*., 1994; Krijgsheld *et al*., 2013). Additionally, asexual development has been studied in *F. graminearum*. In this fungus, only AbaA and FlbD, but not the FlbC homolog, were required for macroconidia formation (Son *et al*., 2013, 2014a). Indeed in *F. fujikuroi*, Aba1 is essential for both micro‐ and macroconidia formation in the strains IMI58289 and E282 respectively.

Furthermore, a microscopic analysis revealed that *F. fujikuroi* Wet1 is likely involved in conidia maturation, as already established for the *A. nidulans* and *F. graminearum* WetA homologs. Thus, *Aspergillus* WetA affects the conidial cell wall composition as well as trehalose accumulation (Sewall *et al*., 1990; Tao and Yu, 2011), while *F. graminearum* Δ*wetA* mutants had malformed and longer conidia with fewer septa that were sensitive to stressors (Son *et al*., 2014b). Although the exact role of *F. fujikuroi* Wet1 remains to be elucidated, one can deduce a conserved role of this protein in conidia separation and maturation for all three filamentous fungi.

We propose a working model in which different global regulators affect *ABA1* expression, and therefore we suggest that Aba1 is the central conidiation‐specific TF in *F. fujikuroi*. Flb3 and Flb4 are positive regulators, while Csm1 is a repressor of *ABA1* expression. Only Flb3 cannot be overruled by Csm1, so that this TF is strictly required for *ABA1* expression (Fig. 9D). Of course, the time‐dependent expression of these regulator genes was not taken into consideration, so that a larger hierarchical network cannot be excluded at this point.

Finally, we identified Set1 as activator and Kdm5 as repressor of *ABA1* expression. Noteworthy, conidiation was not affected upon deletion of *F. graminearum* and *F. verticillioides SET1* (Liu *et al*., 2015; Gu *et al*., 2017). However, in *F. fujikuroi*, conidia formation was nearly abolished in Δ*set1* and increased in Δ*kdm5*, respectively, which correlated with down‐ and upregulated H3K4me3 levels at the 5′ end of *ABA1*.

In conclusion, we present a comprehensive analysis of the H3K4‐specific histone modifiers Set1 and Kdm5 in the rice pathogen *F. fujikuroi*. We show that they antagonize H3K4me3 genome‐wide, affecting genes of primary and secondary metabolism as well as asexual conidia formation. While their influence on subtelomeric SM gene clusters remains to be resolved in the future, a direct regulation of the conidiation‐specific TF gene *ABA1* could be suggested, giving a direct link between H3K4me3 levels, *ABA1* expression and conidia formation.

## Experimental procedures

### *Fungal strains, media and growth conditions*

*F. fujikuroi* IMI58289 (Commonwealth Mycological Institute, Kew, UK) (Wiemann *et al*., 2013) was used as WT strain for the generation of deletion, complementation, point‐mutation and overexpression mutants. Furthermore, *ABA1* was deleted in *F. fujikuroi* E282 (Niehaus *et al*., 2017a).

Vegetative growth was assessed in triplicates on solid V8 (30 mM CaCO_3_, 20%, v/v, vegetable juice; Campbell Food, Puurs, Belgium), CM (Pontecorvo *et al*., 1953), CD (Czapek Dox; Sigma‐Aldrich, Steinheim, Germany) and ICI + 6 mM NH_4_NO_3_ (Imperial Chemical Industries Ltd., London, UK) (Geissman *et al*., 1966) media after incubation for 7 days in the dark (28°C). Furthermore, conidiation was assessed on V8 agar in the presence of a 12 h light/12 h dark cycle (18°C) after incubation for 3 or 14 days for ChIP, gene expression or conidia formation respectively. Light microscopy with an Axio Scope.A1 (Carl Zeiss, Jena, Germany) was performed using colonies grown on conidiation‐inducing KCl plates (8 g l^−1^ KCl, 15 g l^−1^ agar) for 10 days in the dark (28°C). For the isolation of DNA and RNA from solid cultures, the WT and mutant strains were incubated on solidified medium covered with a layer of cellophane.

For submersed cultures, a pre‐culture was done, consisting of 100 ml Darken medium (Darken *et al*., 1959) in 300 ml‐Erlenmeyer flasks, which was shaken at 180 rpm and 28°C for 3 days in the dark. Then, 0.5% (v/v) of this pre‐culture was transferred to the main culture, consisting of 100 ml ICI medium with the appropriate nitrogen source (6 or 60 mM Gln to gain acidic, 6 mM NaNO_3_ to gain alkaline pH conditions) in 300 ml‐Erlenmeyer flasks, being shaken under above described conditions for 3 or 7 days for ChIP, gene expression or SM analyses respectively. Protoplast transformation of *F. fujikuroi* was achieved using a main culture of 100 ml ICI medium with 10 g l^−1^ fructose instead of glucose as well as 0.5 g l^−1^ (NH_4_)_2_SO_4_ as nitrogen source, which was shaken for a maximum of 16 h under above described conditions.

### *Plasmid constructions*

Yeast recombinational cloning (Colot *et al*., 2006; Schumacher, 2012) was applied for the generation of deletion, complementation, point‐mutation and overexpression vectors. For deletion vectors, about 1 kb large fragments upstream and downstream of the respective gene of interest were amplified, 5′ flanks with 5F/5R and 3′ flanks with 3F/3R primer pairs (Supporting Information Table S3). The hygromycin B resistance cassette *hphR* (hygromycin B phosphotransferase gene under the control of the *trpC* promoter from *A. nidulans*) was amplified with hph_F/hph_R (Supporting Information Table S3) from the template pCSN44 (Staben *et al*., 1989). Additionally for *CSM1* deletion, the nourseothricin resistance cassette *natR* (nourseothricin resistance gene under control of *A. nidulans* P*trpC*) was gained from the template pZPnat1 (GenBank AY631958). *S. cerevisiae* FY834 (Winston *et al*., 1995) was transformed with the obtained PCR products (5′ flank, 3′ flank, resistance cassette) as well as with the EcoRI/XhoI digested shuttle vector pRS426 (Christianson *et al*., 1992), gaining the respective deletion vectors.

*SET1* complementation and point‐mutation vectors were generated as follows. The full‐length gene *SET1* including its 5′ sequence was amplified in two overlapping fragments with primer pairs set1_5F/set1_c_R1 and set1_c_F2/set1_c_R2 (Supporting Information Table S4). For the point‐mutated variant, three overlapping fragments were generated to insert the base pair substitutions using primer pairs set1_5F/set1_c_R1, set1_c_F2/set1_mut_R and set1_mut_F/set1_c_R2 (Supporting Information Table S4). The *Tgluc* terminator sequence was amplified from *Botrytis cinerea* B05.10 genomic DNA with BcGlu_Term_F2/Tgluc_Nat1_R (Supporting Information Table S4), while the resistance cassette *natR* and the *SET1* 3′ flank were generated as described above. Then, *S. cerevisiae* FY834 was transformed with the obtained fragments and with the EcoRI/XhoI digested plasmid pRS426, yielding p*SET1* ^*C*^ and p*SET1* ^*H1191K*^ (Supporting Information Fig. S6A).

For overexpression of *KDM5*, the first 1.7 kb of this gene was amplified with OE_kdm5_F/OE_kdm5_R (Supporting Information Table S4) and was cloned into the NcoI/NotI restricted plasmid pNDH‐OGG (Schumacher, 2012), gaining pOE::*KDM5* (Supporting Information Fig. S6B). All complementation, point‐mutation and overexpression vectors were verified by sequencing using primers listed in Supporting Information Table S4.

### *Fungal transformations and analysis of transformants*

*Fusarium fujikuroi* protoplast transformation was carried out as described earlier (Tudzynski *et al*., 1999). Deletion cassettes were amplified from the deletion vectors with primer pairs 5F/3R (Supporting Information Table S3), while 10–40 μg of the ApaI/XbaI digested vectors p*SET1* ^*C*^ and p*SET1* ^*H1191K*^ (Supporting Information Fig. S6A) as well as of the circular vector pOE::*KDM5* (Supporting Information Fig. S6B) were applied for transformation. The transformants were selected with 100 μg ml^−1^ hygromycin B (Calbiochem, Darmstadt, Germany) and/or 100 μg ml^−1^ nourseothricin (Werner‐Bioagents, Jena, Germany) resistance markers.

Single and double deletion mutants were verified by Southern blot and/or diagnostic PCR, showing the homologous integration of deletion cassettes as well as the absence of WT genes. Diagnostic PCRs for three independent Δ*set1* and three independent Δ*kdm5* mutants can be found in Supporting Information Figs. S7A and S8A respectively. Furthermore, Supporting Information Fig. S9A–G shows diagnostic PCRs for two Δ*aba1* (IMI58289 WT background), two E282/Δ*aba1*, two Δ*flb2*, three Δ*flb3*, two Δ*flb4*, three Δ*flb5* and three Δ*wet1* mutants. And Supporting Information Fig. S10A–C depicts diagnostic PCRs for two Δ*aba1*/Δ*csm1*, one Δ*flb3*/Δ*csm1* and two Δ*flb4*/Δ*csm1* double mutants.

Moreover, the *in loco* integration of *SET1* ^*C*^ and *SET1* ^*H1191K*^ constructs was verified with the amplification of 5′ and 3′ flanks and the absence of untransformed nuclei for three independent transformants each (Supporting Information Fig. S6C), and the *in loco* integration of pOE::*KDM5* was shown for three independent transformants (Supporting Information Fig. S6D). A northern blot expression analysis verified the successful overexpression of *KDM5* in the mutants (Supporting Information Fig. S6E). For the complementation and point‐mutation mutants, it was checked that they were unable to grow on hygromycin B (deletion phenotype), but were only able to grow on nourseothricin (complementation phenotype), while the point‐mutation in *SET1* ^*H1191K*^ mutants was verified by sequencing.

### *DNA analysis* via *Southern blot and PCR*

Plasmid DNA from *S. cerevisiae* FY834 and *E. coli* Top10F’ (Invitrogen, Darmstadt, Germany) was isolated using the NucleoSpin Plasmid Kit (Macherey‐Nagel, Düren, Germany). Moreover, *F. fujikuroi* genomic DNA from lyophilised and ground mycelium was extracted as described elsewhere (Cenis, 1992). The analysis of ectopically integrated deletion cassettes via Southern blot (Southern, 1975) started with the digestion of genomic DNA of WT and deletion mutants with an appropriate restriction enzyme (Thermo Fisher Scientific, Schwerte, Germany). The DNA was separated in a 1% (w/v) agarose gel, transferred to a nylon membrane (Nytran SPC; Whatman, Sanford, FL) via downward alkali blotting (Ausubel *et al*., 1987) and hybridized with ^32^P‐labelled probes. Probes were generated with the random oligomer‐primer method (Sambrook *et al*., 1989) using gene flanks as templates (Supporting Information Table S3). Southern blots for Δ*set1* and Δ*kdm5* mutants can be found in Supporting Information Figs. S7B/C and S8B/C respectively. For the amplification of DNA by PCR, BioTherm DNA Polymerase (GeneCraft, Lüdinghausen, Germany), TaKaRa LA Taq DNA Polymerase (Takara Bio, Saint‐Germain‐en‐Laye, France) or Phusion High‐Fidelity DNA Polymerase (Finnzymes, Vantaa, Finland) were used.

### *Expression analysis* via *northern blot and qPCR*

RNA from lyophilised and ground mycelium was extracted with the TRI Reagent (Sigma‐Aldrich, Steinheim, Germany). For northern blot expression analysis (Church and Gilbert, 1984) of OE::*KDM5* mutants, 20 μg of total RNA was separated in a 1% (w/v) denaturating agarose gel (Sambrook *et al*., 1989). Blotting and hybridisation were done as described above. For probe generation, the *KDM5* WT fragment (kdm5_WT_F/kdm5_WT_R; Supporting Information Table S3) was used as template.

For expression analysis via qPCR, 1 μg of DNase I‐treated (Thermo Fisher Scientific, Schwerte, Germany) total RNA was transcribed into cDNA with oligo dT primers and SuperScript II Reverse Transcriptase (Invitrogen, Darmstadt, Germany). Furthermore, iQ SYBR Green Supermix (Bio‐Rad, München, Germany) was used for qPCRs in a C1000 Touch Thermal Cycler with a CFX96 Real‐Time System (Bio‐Rad, München, Germany). Expression levels of *ABA1*, *FLB3*, *FLB4* and *WET1* as well as of the constitutively expressed reference genes (*FFUJ_07710*, GDP mannose transporter gene *GMT*; *FFUJ_05652*, related actin gene *RAC*; *FFUJ_08398*, ubiquitin gene *UBI*) were determined in triplicates or quadruplicates with primer pairs listed in Supporting Information Table S5. Annealing temperatures of 60°C yielded primer efficiencies between 90% and 110% and the expression levels were calculated with the ΔΔCt‐method (Pfaffl, 2001).

### *Expression analysis* via *microarray*

The WT, Δ*set1* and Δ*kdm5* mutants were grown in liquid ICI medium with 6 or 60 mM Gln for 3 days in duplicates. After total RNA isolation as described above, an additional clean‐up step was done with the NucleoSpin RNA Clean‐up Kit (Macherey‐Nagel, Düren, Germany). Agilent Technologies (Santa Clara, CA) designed the microarrays, while Arrows Biomedical (Münster, Germany) prepared the hybridisation according to the manufacturer's protocol. For the heatmaps, the eight expression profiles were extracted first, and were then clustered in a second step using the programme Perseus 1.5.8.5 (Max Planck Institute of Biochemistry, Martinsried, Germany) (Tyanova *et al*., 2016) with standard settings. Genes upregulated in the mutants compared with the WT showed a log_2_ fold change of ≥ 2 (green), downregulated genes of ≤ −2 (red).

The Functional Catalogue (FunCat) for protein sequences (Ruepp *et al*., 2004) was used to identify significantly enriched protein functions. To correct for multiple comparisons in multiple hypotheses testing for the 845 taxonomically allowed FunCat groups, we calculated the Bonferroni correction as well as the false discovery rate with an experiment‐wide significance level of 0.05. The microarray data as well as additional information on sample preparation and data processing are available at the NCBI Gene Expression Omnibus under the accession number GSE90948.

### *Western blot analysis*

The WT, Δ*set1*, Δ*kdm5* and OE::*KDM5* mutants were grown in liquid ICI medium with 60 mM Gln for 3 days. Western blot detection of K4‐methylated H3 proteins in a whole‐protein extract was done as described elsewhere (Janevska *et al*., 2018). The following primary antibodies were used (Active Motif, La Hulpe, Belgium): anti‐H3K4me1 (#39298; 1:10 000), anti‐H3K4me2 (#39141; 1:10 000), anti‐H3K4me3 (#39159; 1:10 000) and anti‐H3 C‐terminal (#39163; 1:10 000). Donkey anti‐rabbit IgG‐HRP served as secondary antibody (sc‐2317; 1:10 000; Santa Cruz Biotechnology, Heidelberg, Germany).

### *ChIP analysis*

For SM‐related genes, the WT, Δ*set1*, Δ*kdm5* and OE::*KDM5* were grown in liquid ICI medium with 6 mM for 3 days. For conidiation‐related genes, the WT, Δ*set1* and Δ*kdm5* were incubated on solid V8 agar with cellophane in the presence of a 12 h light/12 h dark cycle for 3 days. Crosslinking was performed with 1% (v/v) formaldehyde for 15 min at 28°C and 90 rpm, followed by quenching with 125 mM glycine for 5 min at 37°C. Next, the mycelium was separated from the liquid, was shock‐frozen with liquid nitrogen and ground to a fine powder. Further sample preparation was essentially performed as described elsewhere (Gacek‐Matthews *et al*., 2015) using the Bioruptor Plus (Diagenode, Seraing, Belgium) for sonication. The ChIPs were done with the anti‐H3K4me2 (#39141; Active Motif, La Hulpe, Belgium) and anti‐H3K4me3 (#39159; Active Motif, La Hulpe, Belgium) antibodies, followed by treatment with Dynabeads Protein A (Thermo Fisher Scientific, Schwerte, Germany) to precipitate the chromatin‐antibody‐conjugate. ChIP‐qPCR of *CPS/KS*, *P450‐1*, *P450‐2*, *P450‐4*, *ABA1*, *FLB3* and *FLB4* genes was performed in quadruplicates with primers that bind at the 5′ gene ends (Supporting Information Table S5).

### *Chemical analysis of SM production levels*

The WT, Δ*set1*, Δ*kdm5* and OE::*KDM5* mutants were grown in liquid ICI medium with 6 mM Gln (BIK, GA_3_), 6 mM NaNO_3_ (FSR) or 60 mM Gln (FUS, FSA) for 7 days in triplicates. BIK, FSR, FUS and FSA were directly analysed after filter‐sterilization of the supernatant to remove the mycelium (0.45 μm membrane filters; BGB Analytik, Schloßböckelheim, Germany). The four SMs were measured via HPLC‐DAD which was essentially performed as described elsewhere (Studt *et al*., 2012), using a VWR Hitachi Chromaster HPLC system (VWR International, Darmstadt Germany) with an EZChrome Elite 3.3.2 SP2 software (Agilent Technologies, Santa Clara, CA). GA_3_ was extracted and concentrated from 20 ml supernatant applying Sep‐Pak C_18_ cartridges (Waters, Eschborn, Germany) and 2 ml 55% (v/v) acetonitrile for elution. The HPLC‐DAD analysis of GA_3_ was essentially performed as described earlier (Wiemann *et al*., 2012) using the same HPLC system from above. In each case, the production of the SMs was related to the dry weight of the strains which was determined after harvesting and freeze‐drying.

SM extraction and subsequent quantification of GA_3_ levels *in planta* via HPLC coupled to tandem mass spectrometry (HPLC‐MS/MS) were performed as described previously (Studt *et al*., 2017). Mock infection was achieved through the addition of H_2_O and served as negative control. Quantified metabolite levels were normalized to the input sample weights.

### *Rice virulence assay*

The infection of surface‐sterilized and pre‐germinated seedlings of *Oryza sativa* spp. *japonica* cv. Nipponbare (kindly provided by the USDA ARS Dale Bumpers National Rice Research Center, Arkansas, USA) with mycelial plugs of the WT, Δ*set1*, Δ*kdm5* and OE::*KDM5* mutants was performed for 7 days as described elsewhere (Janevska *et al*., 2017). Addition of 100 ppm GA_3_ and H_2_O served as positive and negative control respectively.

## Supporting information


**Fig. S1. Western blot analysis of the Δ*kdm5* mutant**. The western blot was done using the H3K4me3 and H3K4me2 antibodies. Indicated strains were grown in liquid culture (ICI + 60 mM Gln) for 3 days prior to protein extraction. 15 μg of the protein extract was loaded on to the gel, and an unspecific band served as loading control.
**Fig. S2. Microarray expression analysis of differentially regulated A) bikaverin (BIK) and B) apicidin F (APF) cluster genes in Δ*set1* and Δ*kdm5***. The WT and the two deletion mutants were grown in liquid culture (ICI + 60 mM Gln) for 3 days prior to RNA extraction. Data are mean values (*n* = 2). Genes upregulated in the deletion mutants compared with the WT are green (significant when log_2_ fold change ≥2), downregulated genes are red (significant when log_2_ fold change ≤ −2), and not differentially expressed genes are white (between −2 and 2). The tables show the gene accession numbers and the respective cluster genes, while non‐cluster genes are highlighted in grey. The cluster organization is depicted schematically below, with arrows indicating the direction of transcription and white bars indicating introns.
**Fig. S3. Chromosomal location of the analysed SM gene clusters**. The distribution of H3K27me3 is shown for A) chromosome II, B) chromosome III, C) chromosome V, and D) chromosome IX (taken from Studt *et al*., 2016b). The location of the respective SM key genes is indicated on the chromosomes. Furthermore, the distribution of H3K4me2 is shown for the gibberellic acid (GA) gene cluster (taken from Wiemann *et al*., 2013). For both ChIP‐Seq analyses, the WT was grown in liquid culture (ICI + 6 mM Gln) for 3 days.
**Fig. S4. Virulence on rice of Δ*set1*, Δ*kdm5* and OE::*KDM5* mutants**. Germinated rice seedlings were infected with 100 ppm gibberellic acid GA_3_ (positive control), H_2_O (negative control), and indicated strains for 7 days. Data are mean values ±SD (*n* = 3). For statistical analysis, the mutants were compared with the WT using the student's *t*‐test: **, *P* < 0.01.
**Fig. S5. Plate assay and microscopic analysis of Δ*aba1*, Δ*flb2*‐Δ*flb5* and Δ*wet1* mutants**. A) Indicated strains were grown on V8 agar in the presence of a 12 h light/12 h dark cycle for 14 days to induce conidiation. B) Microscopic analysis of microconidia formation in the *F. fujikuroi* WT IMI58289 and the respective Δ*wet1* deletion mutant grown on KCl agar for 10 days. For both strains, the same microscopic magnification was applied.
**Fig. S6. Generation of *SET1*** ^***C***^, ***SET1*** ^***H1191K***^ **and OE::*KDM5* mutants**. A) The deletion mutant Δ*set1* T34 was transformed with ApaI/XbaI digested p*SET1* ^*C*^ or p*SET1* ^*H1191K*^ vectors harbouring the WT or point‐mutated *SET1* gene, respectively, driven by its native promoter as well as the nourseothricin resistance cassette *natR*. The nucleotide substitutions resulting in the point mutation are indicated. B) *KDM5* was overexpressed with the constitutive *PoliC* promoter from *A. nidulans*. The first 1.7 kb of *KDM5* was cloned into NcoI/NotI restricted pNDH‐OGG conferring hygromycin B resistance (*hphR*). C) The *in loco* integration of the constructs in three independent *SET1* ^*C*^ and *SET1* ^*H1191K*^ mutants, respectively, was verified using primer pairs set1_5diag/set1_c_diag (5′ flank; 1.40 kb), set1_3diag/nat1_R1 (3′ flank; 1.57 kb) and set1_5diag/trpC_T (untransformed nuclei; 1.25 kb for Δ*set1*). The respective deletion mutant (Δ) and the WT were used as controls. D) The *in loco* integration of pOE::*KDM5* in the three transformants was checked using primer pair PoliC_Seq_F2/OE_kdm5_diag (1.95 kb). M, GeneRuler DNA Ladder Mix. E) The WT and OE::*KDM5* mutants were grown in liquid culture (ICI + 60 mM Gln) for 3 days prior to RNA extraction from the harvested mycelium. The northern blot was probed with DNA corresponding to *KDM5*, and the ribosomal RNA was visualized for the respective gel as loading control.
**Fig. S7. Verification of Δ*set1* deletion mutants by diagnostic PCR and Southern blot**. A) Deletion via homologous recombination with the hygromycin B resistance cassette (*hphR*) was verified with the amplification of 5′ (set1_5diag/trpC_T) and 3′ (set1_3diag/trpC_P2) flanks but no amplification of WT (set1_WT_F/set1_WT_R) signal for three independent transformants. B) For analysing ectopic integration of deletion constructs, genomic DNA of transformants and WT was digested with ClaI, while the 5′ flank was applied for probing. C) Detected signals match the expected 10.66 kb for the WT and 3.11 kb for Δ*set1*. λ, λ/HindIII; M, GeneRuler 1 kb Plus DNA Ladder.
**Fig. S8. Verification of Δ*kdm5* deletion mutants by diagnostic PCR and Southern blot**. A) Deletion via homologous recombination with the hygromycin B resistance cassette (*hphR*) was verified with the amplification of 5′ (kdm5_5diag/trpC_T) and 3′ (kdm5_3diag/trpC_P2) flanks but no amplification of WT (kdm5_WT_F/kdm5_WT_R) signal for three independent transformants. B) For analysing ectopic integration of deletion constructs, genomic DNA of transformants and WT was digested with SalI, while the 3′ flank was applied for probing. C) Detected signals match the expected 7.07 kb for the WT and 2.70 kb for Δ*kdm5*. λ, λ/HindIII; M, GeneRuler DNA Ladder Mix.
**Fig. S9. Verification of Δ*aba1*, Δ*flb2*‐Δ*flb5* and Δ*wet1* single deletion mutants**. Deletion via homologous recombination with the hygromycin B resistance cassette was verified with the amplification of 5′ (5diag/trpC_T) and 3′ (3diag/trpC_P2) flanks but no amplification of WT (WT_F/WT_R) signal for A) two independent Δ*aba1* mutants (IMI58289 WT background), B) two independent E282/Δ*aba1* mutants, C) two independent Δ*flb2* mutants, D) three independent Δ*flb3* mutants, E) two independent Δ*flb4* mutants, F) three independent Δ*flb5* mutants and G) three independent Δ*wet1* mutants. λ, λ/HindIII.
**Fig. S10. Deletion of *CSM1* in Δ*aba1*, Δ*flb3* and Δ*flb4* backgrounds**. Deletion of *CSM1* via homologous recombination with the nourseothricin resistance cassette was verified with the amplification of 5′ (csm1_5diag/nat1_hiF) and 3′ (csm1_3diag/trpC_P2) flanks but no amplification of WT (csm1_WT_F/csm1_WT_R) signal for A) two independent Δ*aba1*/Δ*csm1* double mutants, B) one Δ*flb3*/Δ*csm1* double mutant and C) two independent Δ*flb4*/Δ*csm1* double mutants. λ, λ/HindIII.
**Table S1. Significantly enriched protein functions of genes deregulated in Δ*set1* and/or Δ*kdm5* in the microarray expression analysis**. Genes upregulated in the deletion mutants compared with the WT have a log_2_ fold change ≥2, downregulated genes have a log_2_ fold change ≤ −2. The table is sorted by the *P*‐value and the filters rely on the following criteria: B, Bonferroni correction <0.05; F, *P*‐value < FDR; *, *P*‐value <0.05. abs., absolute; rel., relative; FDR, false discovery rate.
**Table S2. Microarray expression analysis of differentially regulated SM key genes in Δ*set1* and Δ*kdm5***. The WT and the two deletion mutants were grown in ICI liquid culture in the presence of limiting (6 mM, N‐) and saturating (60 mM, N+) amounts of Gln for 3 days prior to RNA extraction. Data are mean values (*n* = 2). Genes upregulated in the deletion mutants compared with the WT are green (log_2_ fold change ≥2), and downregulated genes are red (log_2_ fold change ≤ −2). Shown are the gene accession numbers, the encoded SM key genes as well as the produced SMs. PKS, polyketide synthase; NRPS, non‐ribosomal peptide synthetase; STC, sesquiterpene cyclase; DTC, diterpene cyclase; TeTC, tetraterpene cyclase; DMATS, dimethylallyltryptophan synthase.
**Table S3. Primer sequences used for the generation of deletion constructs and for the verification of their homologous integration**. Introduced overhangs required for yeast recombinational cloning are underlined.
**Table S4. Primer sequences used for the generation and analysis of complementation, point‐mutation and overexpression vectors**. Introduced overhangs required for yeast recombinational cloning are underlined.
**Table S5. Primer sequences for analysing relative expression (qPCR) and ChIP‐qPCR (5′ChIP)**. Reference genes for relative expression: *GMT*, GDP mannose transporter gene; *RAC*, related actin gene; *UBI*, ubiquitin gene.
