# Supplementary material for: Set1 and Kdm5 are antagonists for H3K4 methylation and regulators of the major conidiation‐specific transcription factor gene ABA1 in Fusarium fujikuroi
Source: Environ Microbiol. 2018 Sep 18;20(9):3343–62. doi: 10.1111/1462-2920.14339 (PMC6175112; doi:10.1111/1462-2920.14339)
Supplement: Supplementary file 1 — Fig. S1. Western blot analysis of the Δkdm5 mutant. The western blot was done using the H3K4me3 and H3K4me2 antibodies. Indicated strains were grown in liquid culture (ICI + 60 mM Gln) for 3 days prior to protein extraction. 15 μg of the protein extract was loaded on to the gel, and an unspecific band served as loading control. Fig. S2. Microarray expression analysis of differentially regulated A) bikaverin (BIK) and B) apicidin F (APF) cluster genes in Δset1 and Δkdm5. The WT and the two deletion mutants were grown in liquid culture (ICI + 60 mM Gln) for 3 days prior to RNA extraction. Data are mean values (n = 2). Genes upregulated in the deletion mutants compared with the WT are green (significant when log2 fold change ≥2), downregulated genes are red (significant when log2 fold change ≤ −2), and not differentially expressed genes are white (between −2 and 2). The tables show the gene accession numbers and the respective cluster genes, while non‐cluster genes are highlighted in grey. The cluster organization is depicted schematically below, with arrows indicating the direction of transcription and white bars indicating introns. Fig. S3. Chromosomal location of the analysed SM gene clusters. The distribution of H3K27me3 is shown for A) chromosome II, B) chromosome III, C) chromosome V, and D) chromosome IX (taken from Studt et al., 2016b). The location of the respective SM key genes is indicated on the chromosomes. Furthermore, the distribution of H3K4me2 is shown for the gibberellic acid (GA) gene cluster (taken from Wiemann et al., 2013). For both ChIP‐Seq analyses, the WT was grown in liquid culture (ICI + 6 mM Gln) for 3 days. Fig. S4. Virulence on rice of Δset1, Δkdm5 and OE::KDM5 mutants. Germinated rice seedlings were infected with 100 ppm gibberellic acid GA3 (positive control), H2O (negative control), and indicated strains for 7 days. Data are mean values ±SD (n = 3). For statistical analysis, the mutants were compared with the WT using the student's t‐te [file EMI-20-3343-s001.docx]

**Supporting Information:**

**Set1 and Kdm5 are antagonists for H3K4 methylation and regulators of the major conidiation-specific transcription factor gene *ABA1* in *Fusarium fujikuroi***

Slavica Janevska^1^, Ulrich Güldener^2^, Michael Sulyok^3^, Bettina Tudzynski^1^, Lena Studt^1,4†^*

^1^ Institute of Plant Biology and Biotechnology, Westfälische Wilhelms-Universität Münster, 48143 Münster, Germany

^2^ Department of Bioinformatics, TUM School of Life Sciences Weihenstephan, Technical University of Munich, 85354 Freising, Germany

^3^ Center for Analytical Chemistry, Department IFA-Tulln, University of Natural Resources and Life Sciences, Vienna (BOKU), 3430 Tulln, Austria

^4^ Department of Applied Genetics and Cell Biology-Tulln, University of Natural Resources and Life Sciences, Vienna (BOKU), 3430 Tulln, Austria

^†^ Present address: Department of Applied Genetics and Cell Biology-Tulln, University of Natural Resources and Life Sciences, Vienna (BOKU), 3430 Tulln, Austria

* **Corresponding author:** L. Studt, Division of Microbial Genetics and Pathogen Interaction, Department of Applied Genetics and Cell Biology-Tulln, University of Natural Resources and Life Sciences, Vienna (BOKU), Konrad Lorenz-Straße 24, 3430 Tulln, Austria

E-mail: lena.studt@boku.ac.at, phone: +43 1 47654-94193

**Running title**: Set1 and Kdm5 antagonise H3K4me in *F. fujikuroi*


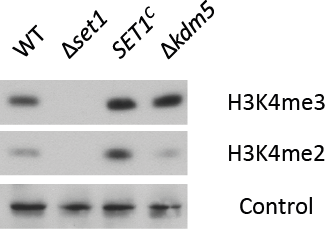


**Figure S1. Western blot analysis of the Δ*kdm5* mutant**. The western blot was done using the H3K4me3 and H3K4me2 antibodies. Indicated strains were grown in liquid culture (ICI+60 mM Gln) for 3 days prior to protein extraction. 15 µg of the protein extract was loaded on to the gel, and an unspecific band served as loading control.


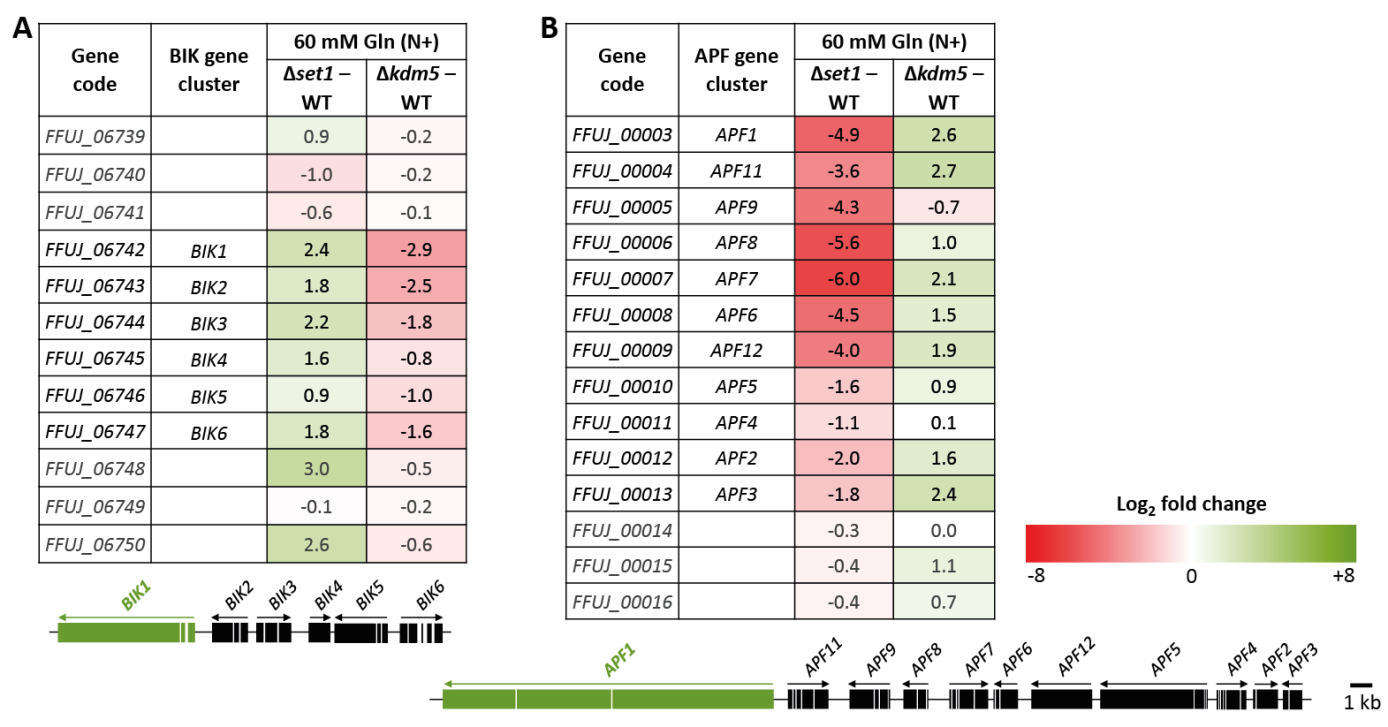


**Figure S2. Microarray expression analysis of differentially regulated A) bikaverin (BIK) and B) apicidin F (APF) cluster genes in Δ*set1* and Δ*kdm5*.** The WT and the two deletion mutants were grown in liquid culture (ICI+60 mM Gln) for 3 days prior to RNA extraction. Data are mean values (*n* = 2). Genes upregulated in the deletion mutants compared to the WT are green (significant when log_2_ fold change ≥ 2), downregulated genes are red (significant when log_2_ fold change ≤ ‑2), and not differentially expressed genes are white (between ‑2 and 2). The tables show the gene accession numbers and the respective cluster genes, while non-cluster genes are highlighted in grey. The cluster organisation is depicted schematically below, with arrows indicating the direction of transcription and white bars indicating introns.


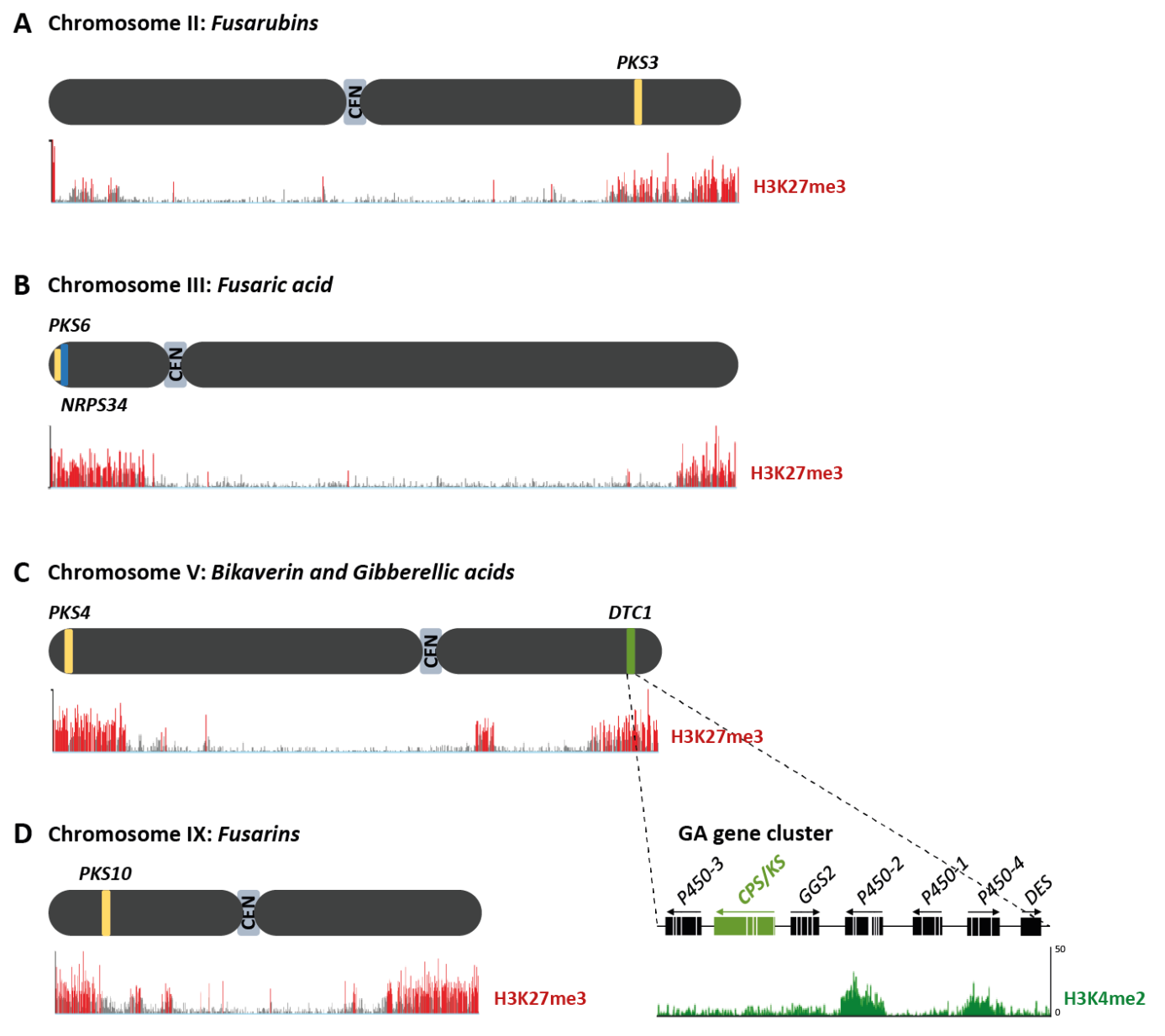


**Figure S3. Chromosomal location of the analysed SM gene clusters.** The distribution of H3K27me3 is shown for A) chromosome II, B) chromosome III, C) chromosome V, and D) chromosome IX (taken from Studt *et al*., 2016b). The location of the respective SM key genes is indicated on the chromosomes. Furthermore, the distribution of H3K4me2 is shown for the gibberellic acid (GA) gene cluster (taken from Wiemann *et al*., 2013). For both ChIP-Seq analyses, the WT was grown in liquid culture (ICI+6 mM Gln) for 3 days.

**
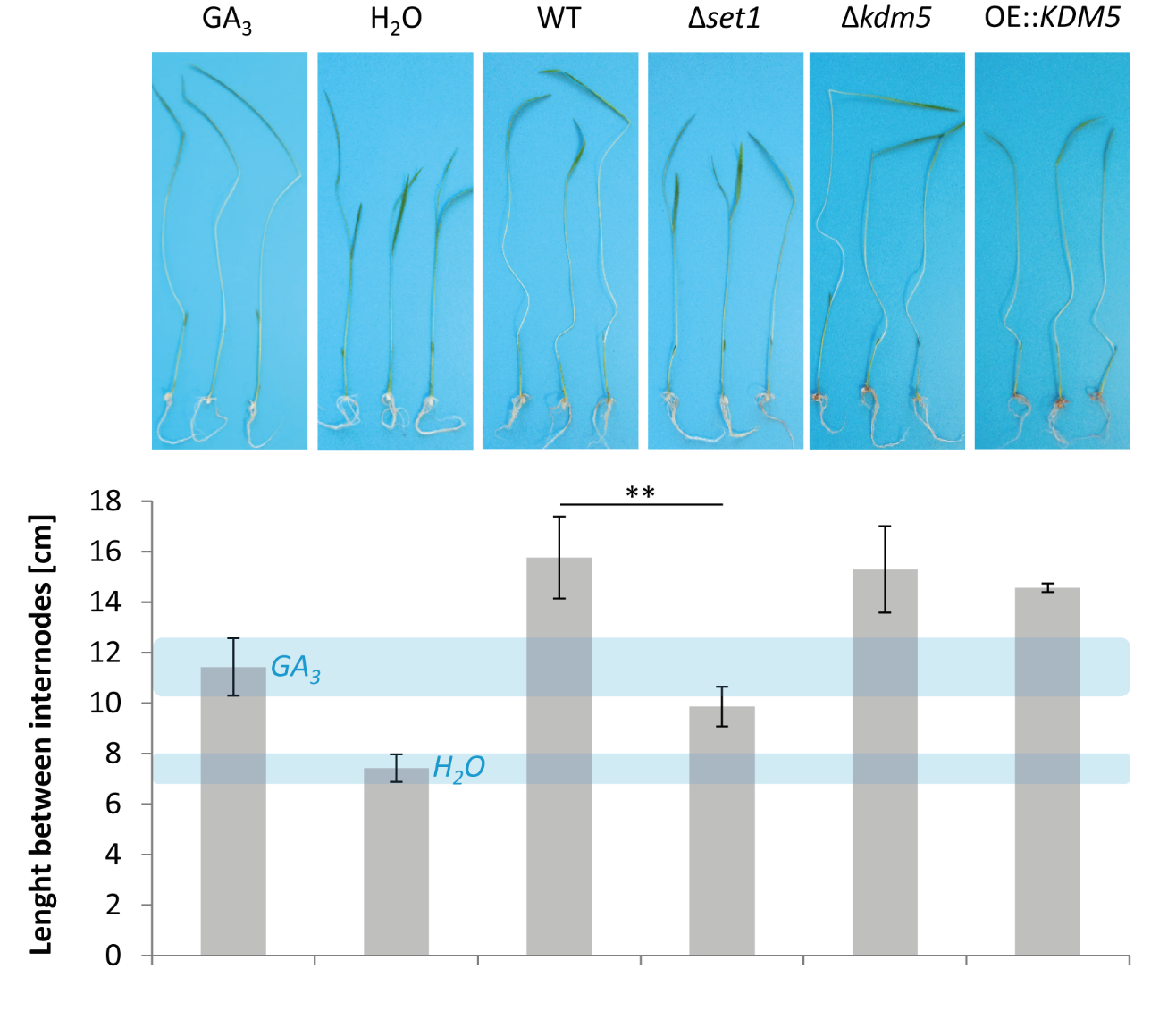
**

**Figure S4. Virulence on rice of Δ*set1*, Δ*kdm5* and OE::*KDM5* mutants.** Germinated rice seedlings were infected with 100 ppm gibberellic acid GA_3_ (positive control), H_2_O (negative control), and indicated strains for 7 days. Data are mean values ± SD (*n* = 3). For statistical analysis, the mutants were compared with the WT using the student’s *t*-test: **, *P* < 0.01.


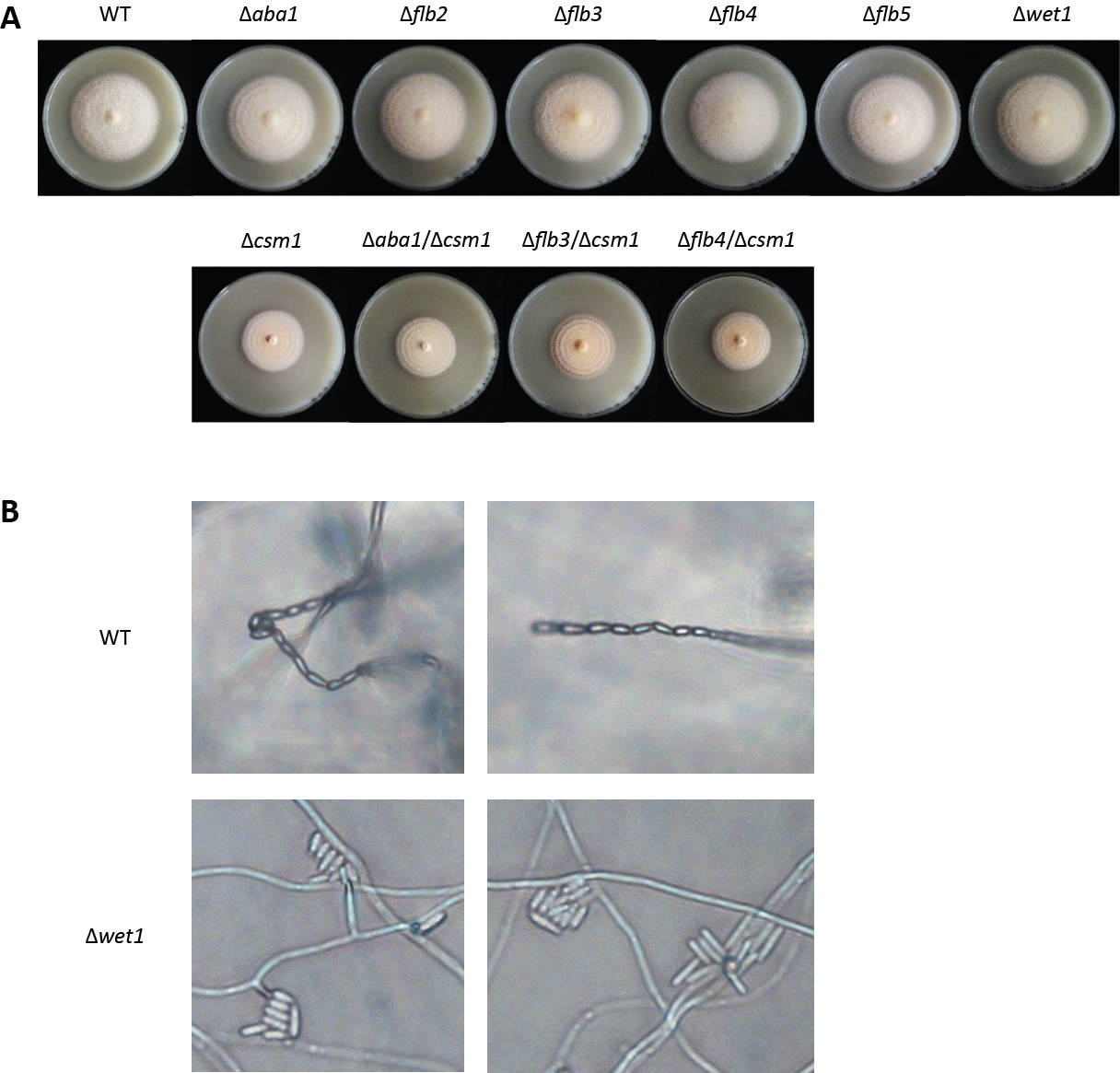


**Figure S5. Plate assay and microscopic analysis of Δ*aba1*, Δ*flb2*-Δ*flb5* and Δ*wet1* mutants.** A) Indicated strains were grown on V8 agar in the presence of a 12 h light/12 h dark cycle for 14 days to induce conidiation. B) Microscopic analysis of microconidia formation in the *F. fujikuroi* WT IMI58289 and the respective Δ*wet1* deletion mutant grown on KCl agar for 10 days. For both strains, the same microscopic magnification was applied.


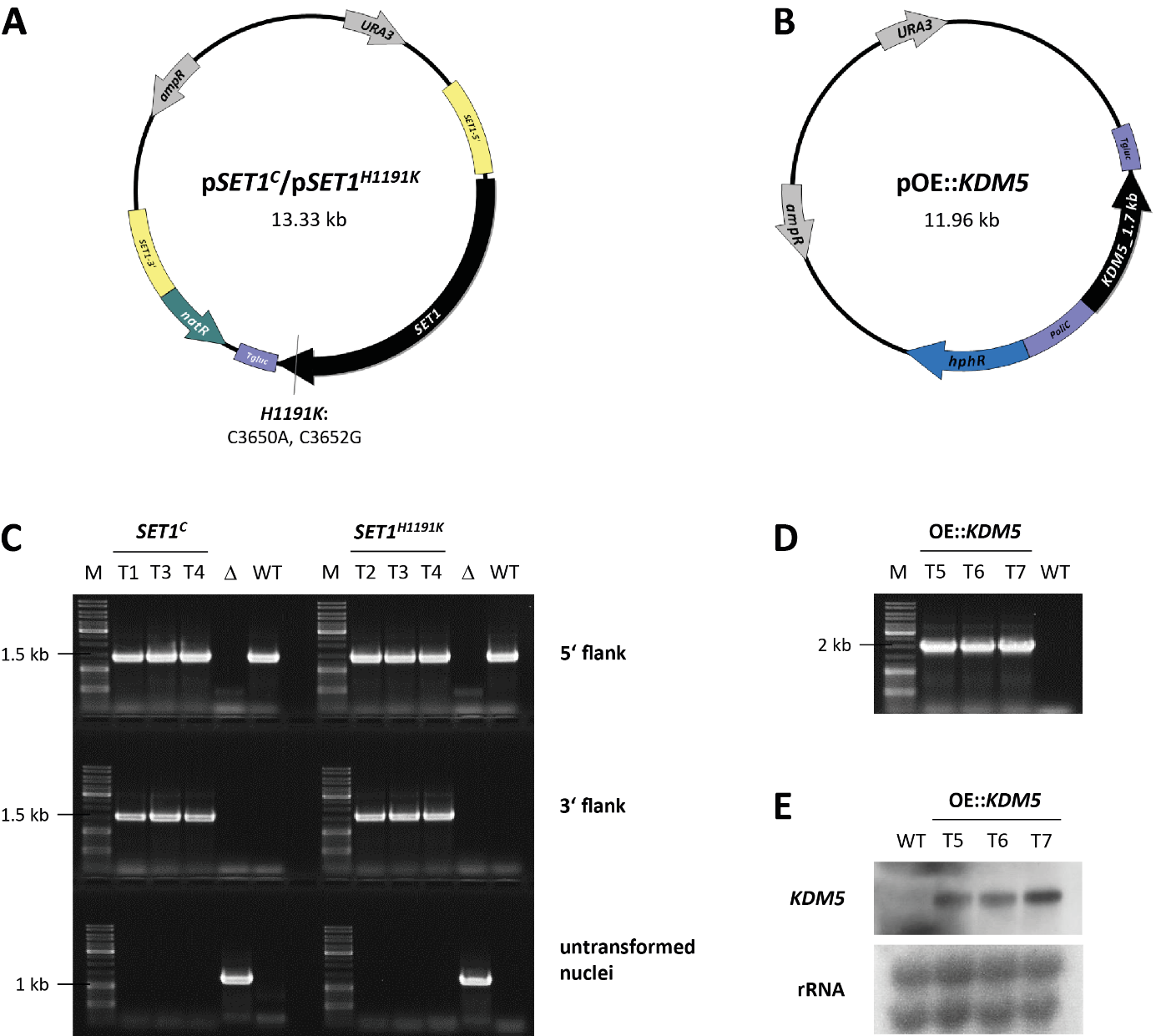


**Figure S6. Generation of *SET1^C^*, *SET1^H1191K^* and OE::*KDM5* mutants.** A) The deletion mutant Δ*set1* T34 was transformed with ApaI/XbaI digested p*SET1^C^* or p*SET1^H1191K^* vectors harbouring the WT or point-mutated *SET1* gene, respectively, driven by its native promoter as well as the nourseothricin resistance cassette *natR*. The nucleotide substitutions resulting in the point mutation are indicated. B) *KDM5* was overexpressed with the constitutive *PoliC* promoter from *A. nidulans*. The first 1.7 kb of *KDM5* was cloned into NcoI/NotI restricted pNDH-OGG conferring hygromycin B resistance (*hphR*). C) The *in loco* integration of the constructs in three independent *SET1^C^* and *SET1^H1191K^* mutants, respectively, was verified using primer pairs set1_5diag/set1_c_diag (5‘ flank; 1.40 kb), set1_3diag/nat1_R1 (3‘ flank; 1.57 kb) and set1_5diag/trpC_T (untransformed nuclei; 1.25 kb for Δ*set1*). The respective deletion mutant (Δ) and the WT were used as controls. D) The *in loco* integration of pOE::*KDM5* in the three transformants was checked using primer pair PoliC_Seq_F2/OE_kdm5_diag (1.95 kb). M, GeneRuler DNA Ladder Mix. E) The WT and OE::*KDM5* mutants were grown in liquid culture (ICI+60 mM Gln) for 3 days prior to RNA extraction from the harvested mycelium. The northern blot was probed with DNA corresponding to *KDM5*, and the ribosomal RNA was visualised for the respective gel as loading control.


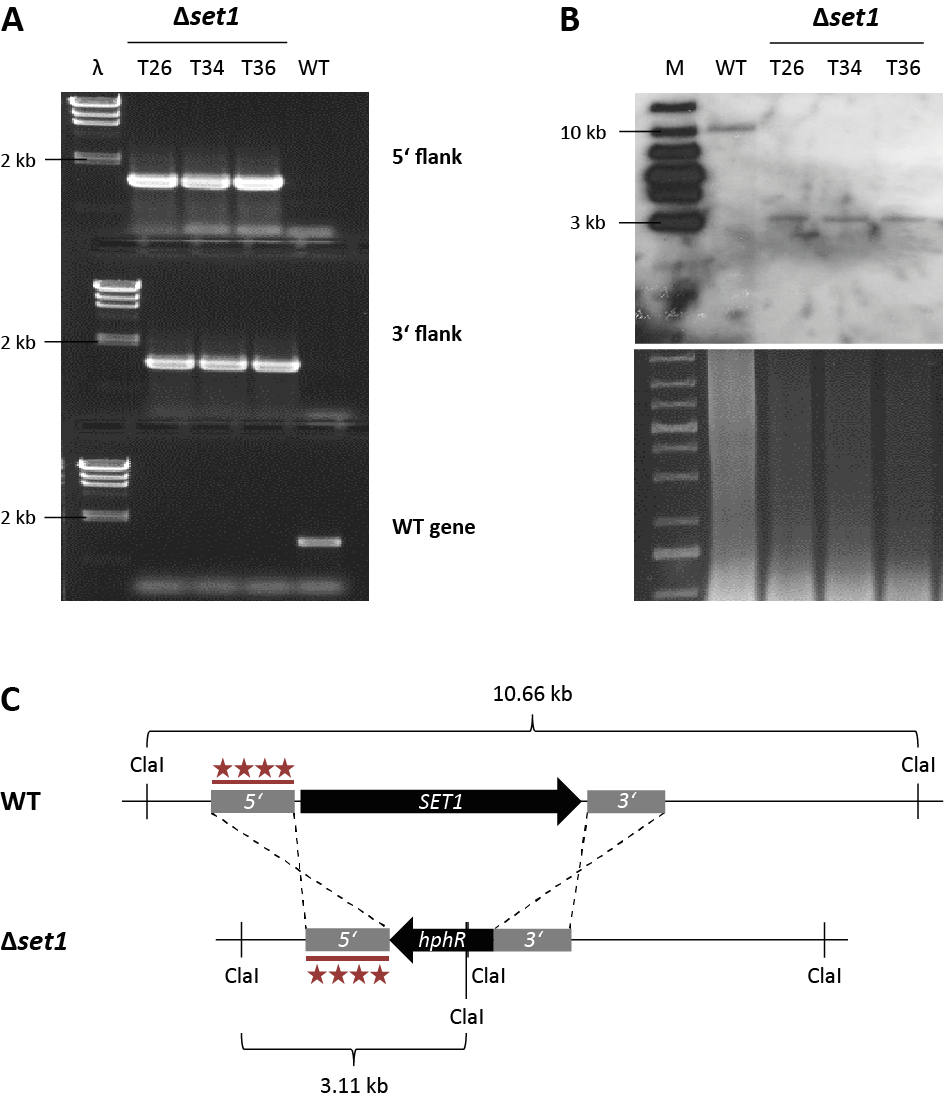


**Figure S7. Verification of Δ*set1* deletion mutants by diagnostic PCR and Southern blot.** A) Deletion *via* homologous recombination with the hygromycin B resistance cassette (*hphR*) was verified with the amplification of 5’ (set1_5diag/trpC_T) and 3’ (set1_3diag/trpC_P2) flanks but no amplification of WT (set1_WT_F/set1_WT_R) signal for three independent transformants. B) For analysing ectopic integration of deletion constructs, genomic DNA of transformants and WT was digested with ClaI, while the 5’ flank was applied for probing. C) Detected signals match the expected 10.66 kb for the WT and 3.11 kb for Δ*set1*. λ, λ/HindIII; M, GeneRuler 1 kb Plus DNA Ladder.


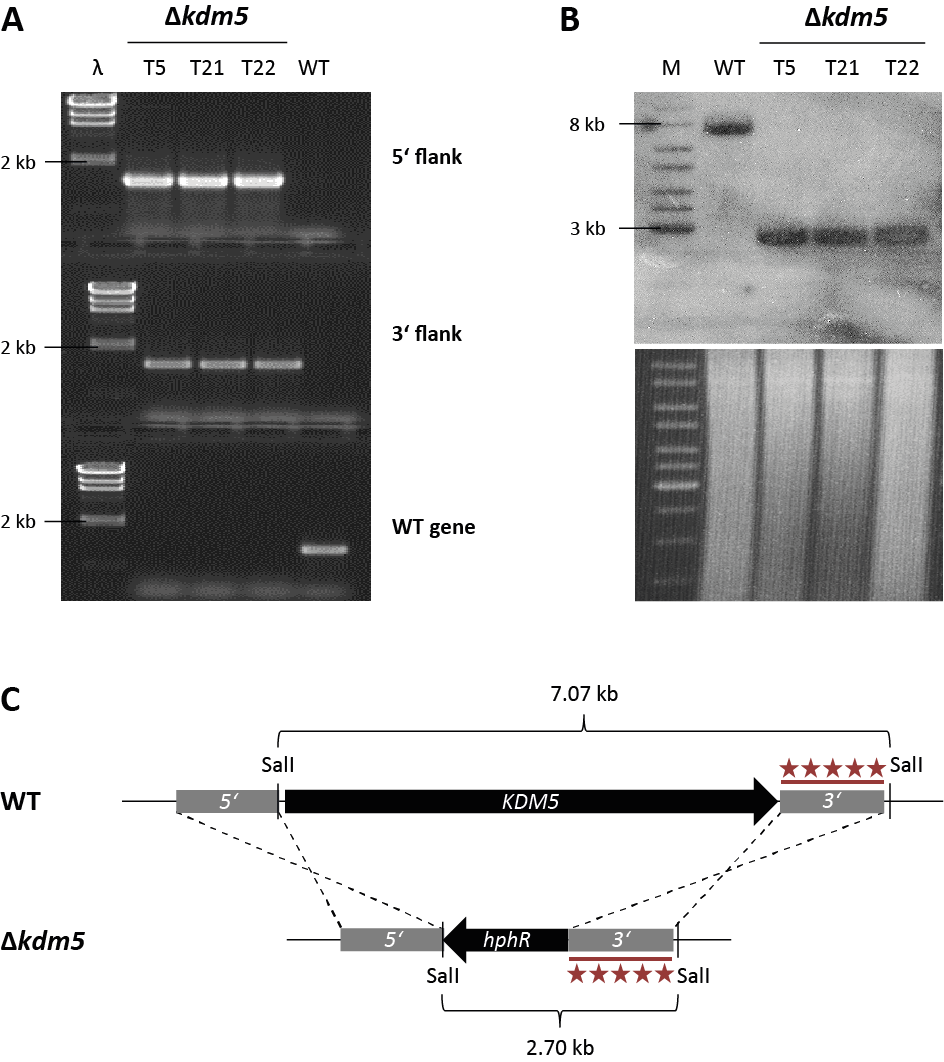


**Figure S8. Verification of Δ*kdm5* deletion mutants by diagnostic PCR and Southern blot.** A) Deletion *via* homologous recombination with the hygromycin B resistance cassette (*hphR*) was verified with the amplification of 5’ (kdm5_5diag/trpC_T) and 3’ (kdm5_3diag/trpC_P2) flanks but no amplification of WT (kdm5_WT_F/kdm5_WT_R) signal for three independent transformants. B) For analysing ectopic integration of deletion constructs, genomic DNA of transformants and WT was digested with SalI, while the 3’ flank was applied for probing. C) Detected signals match the expected 7.07 kb for the WT and 2.70 kb for Δ*kdm5*. λ, λ/HindIII; M, GeneRuler DNA Ladder Mix.


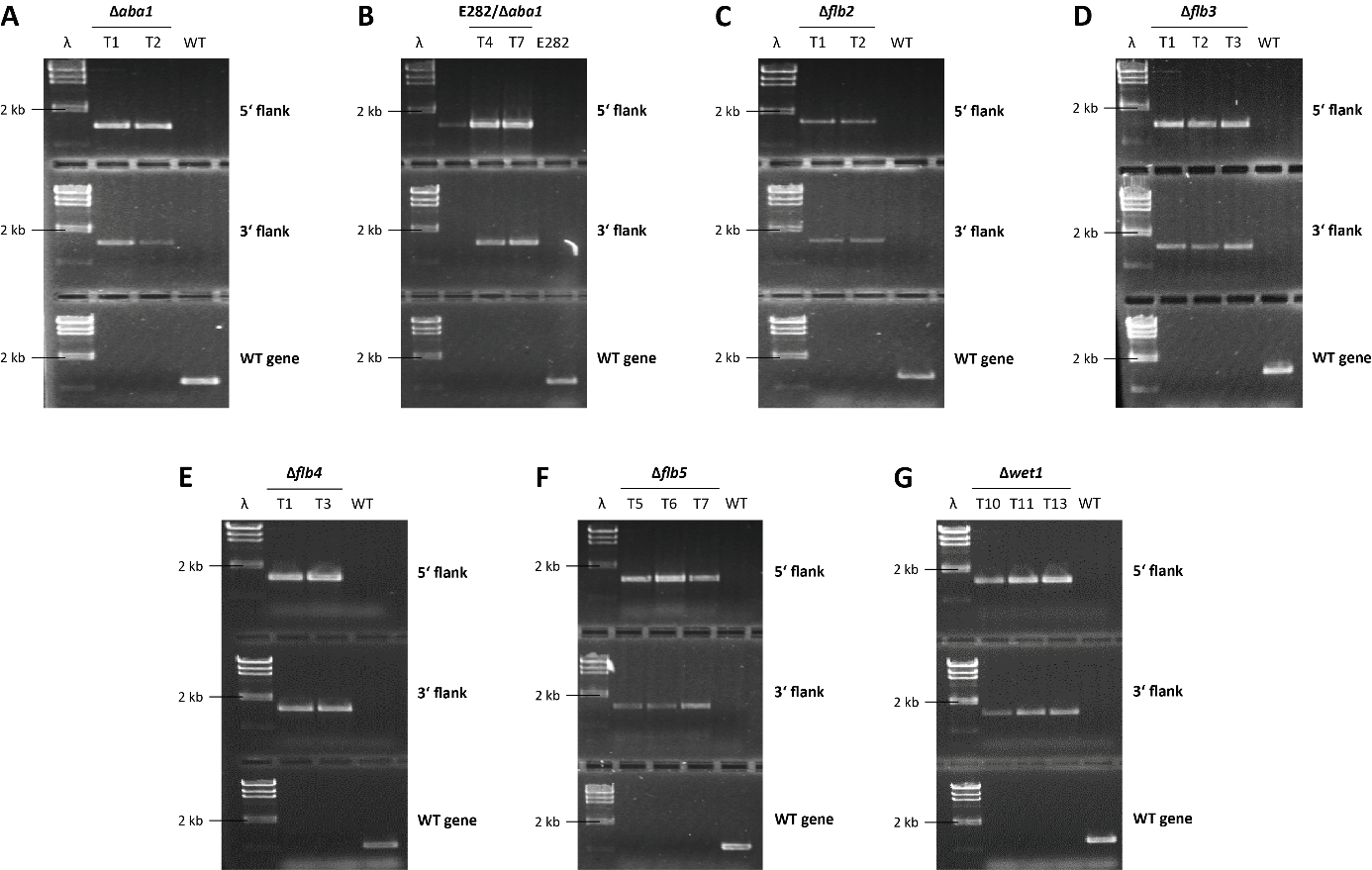


**Figure S9. Verification of Δ*aba1*, Δ*flb2*-Δ*flb5* and Δ*wet1* single deletion mutants.** Deletion *via* homologous recombination with the hygromycin B resistance cassette was verified with the amplification of 5’ (5diag/trpC_T) and 3’ (3diag/trpC_P2) flanks but no amplification of WT (WT_F/WT_R) signal for A) two independent Δ*aba1* mutants (IMI58289 WT background), B) two independent E282/Δ*aba1* mutants, C) two independent Δ*flb2* mutants, D) three independent Δ*flb3* mutants, E) two independent Δ*flb4* mutants, F) three independent Δ*flb5* mutants and G) three independent Δ*wet1* mutants. λ, λ/HindIII.


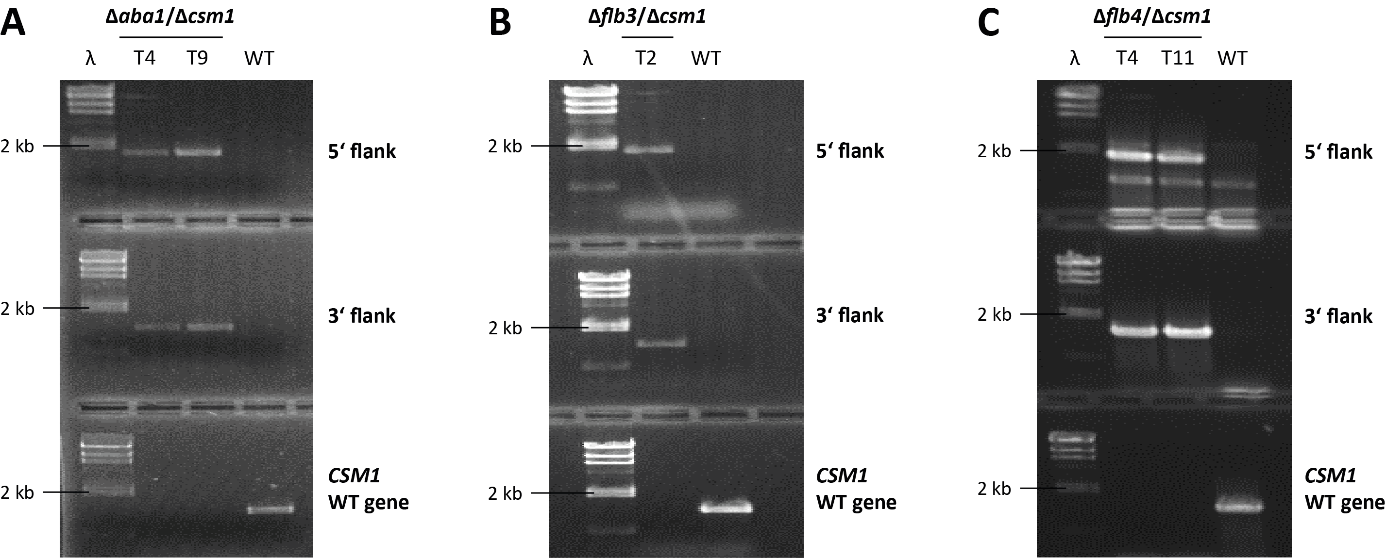


**Figure S10.** **Deletion of *CSM1* in Δ*aba1*, Δ*flb3* and Δ*flb4* backgrounds.** Deletion of *CSM1* *via* homologous recombination with the nourseothricin resistance cassette was verified with the amplification of 5’ (csm1_5diag/nat1_hiF) and 3’ (csm1_3diag/trpC_P2) flanks but no amplification of WT (csm1_WT_F/csm1_WT_R) signal for A) two independent Δ*aba1*/Δ*csm1* double mutants, B) one Δ*flb3*/Δ*csm1* double mutant and C) two independent Δ*flb4*/Δ*csm1* double mutants. λ, λ/HindIII.

**Table S1. Significantly enriched protein functions of genes deregulated in Δ*set1* and/or Δ*kdm5* in the microarray expression analysis.** Genes upregulated in the deletion mutants compared to the WT have a log_2_ fold change ≥ 2, downregulated genes have a log_2_ fold change ≤ ‑2. The table is sorted by the *P*-value and the filters rely on the following criteria: B, Bonferroni correction < 0.05; F, *P*-value < FDR; *, *P*-value < 0.05. abs., absolute; rel., relative; FDR, false discovery rate.

| **Filter** | **FunCat**  **Functional Category** | **Abs. set** | **Rel. set** | **Absolute genome** | **Relative genome** | **Rel. set/ relative genome** | ***P*-value** | **Bonferroni corrected *P*-value** | **FDR** |
| --- | --- | --- | --- | --- | --- | --- | --- | --- | --- |
| **6 mM Gln (N-), Profile 2: upregulated in Δ*set1* in comparison to WT, not affected in Δ*kdm5*** | | | | | | | | | |
| BF* | 01.20 secondary metabolism | 204 | 24.40 | 2127 | 14.30 | 1.71 | 1.49E-15 | 1.26E-12 | 1.26E-12 |
| BF* | 01 metabolism | 365 | 43.60 | 4949 | 33.40 | 1.31 | 1.39E-10 | 1.17E-07 | 5.87E-08 |
| BF* | 01.05 C-compound and carbohydrate metabolism | 166 | 19.80 | 1914 | 12.90 | 1.53 | 4.23E-09 | 3.58E-06 | 1.19E-06 |
| BF* | 02.07 pentose-phosphate pathway | 15 | 1.79 | 75 | 0.50 | 3.58 | 1.57E-05 | 1.32E-02 | 3.31E-03 |
| BF* | 32.07 detoxification | 61 | 7.29 | 621 | 4.19 | 1.74 | 1.58E-05 | 1.34E-02 | 2.67E-03 |
| BF* | 01.06.05 fatty acid metabolism | 26 | 3.11 | 198 | 1.33 | 2.34 | 5.08E-05 | 4.29E-02 | 7.15E-03 |
| F* | 01.20.05 metabolism of acetic acid derivatives | 13 | 1.55 | 75 | 0.50 | 3.10 | 2.62E-04 | 2.21E-01 | 3.16E-02 |
| **60 mM Gln (N+), Profile 2: upregulated in Δ*set1* in comparison to WT, not affected in Δ*kdm5*** | | | | | | | | | |
| BF* | 01.20 secondary metabolism | 178 | 23.00 | 2127 | 14.30 | 1.61 | 2.36E-11 | 2.00E-08 | 2.00E-08 |
| BF* | 01 metabolism | 310 | 40.10 | 4949 | 33.40 | 1.20 | 3.71E-05 | 3.13E-02 | 1.57E-02 |
| **6 mM Gln (N-), Profile 4: downregulated in both Δ*set1* and Δ*kdm5* in comparison to WT** | | | | | | | | | |
| BF* | 01.20 secondary metabolism | 27 | 43.50 | 2127 | 14.30 | 3.04 | 2.48E-08 | 2.10E-05 | 2.10E-05 |
| F* | 34.11.01 photopercep-tion and response | 4 | 6.45 | 58 | 0.39 | 16.54 | 9.97E-05 | 8.42E-02 | 4.21E-02 |

**Table S2. Microarray expression analysis of differentially regulated SM key genes in Δ*set1* and Δ*kdm5*.** The WT and the two deletion mutants were grown in ICI liquid culture in the presence of limiting (6 mM, N-) and saturating (60 mM, N+) amounts of Gln for 3 days prior to RNA extraction. Data are mean values (*n* = 2). Genes upregulated in the deletion mutants compared to the WT are green (log_2_ fold change ≥ 2), and downregulated genes are red (log_2_ fold change ≤ ‑2). Shown are the gene accession numbers, the encoded SM key genes as well as the produced SMs. PKS, polyketide synthase; NRPS, non-ribosomal peptide synthetase; STC, sesquiterpene cyclase; DTC, diterpene cyclase; TeTC, tetraterpene cyclase; DMATS, dimethylallyltryptophan synthase.

| **Gene code** | **Key enzyme** | **6 mM Gln (N-)** | | **60 mM Gln (N+)** | | **WT (60 mM) – WT (6 mM)** | **Product** |
| --- | --- | --- | --- | --- | --- | --- | --- |
|  |  | **Δ*set1* – WT** | **Δ*kdm5* – WT** | **Δ*set1* – WT** | **Δ*kdm5* – WT** |  |  |
| *FFUJ_02219* | PKS1/NRPS | 3.3 | -0.3 | 1.8 | -0.4 | 1.5 | Trichosetin |
| *FFUJ_00118* | PKS2 | -1.0 | 2.2 | -1.1 | -0.5 | 2.2 |  |
| *FFUJ_06742* | PKS4 | 0.7 | 0.7 | 2.4 | -2.9 | -4.0 | Bikaverin |
| *FFUJ_02105* | PKS6 | 1.2 | 0.1 | -1.3 | -2.6 | 11.1 | Fusaric acid |
| *FFUJ_14695* | PKS9/NRPS | 2.8 | 0.6 | -0.4 | 0.3 | 0.8 |  |
| *FFUJ_10058* | PKS10/NRPS | 5.4 | -0.2 | 1.4 | -0.5 | 8.0 | Fusarins |
| *FFUJ_09241* | PKS11 | -6.2 | -5.8 | 0.4 | -0.2 | -4.8 | Fumonisins |
| *FFUJ_12020* | PKS13 | 2.6 | -0.6 | -0.8 | -0.8 | 3.7 | Gibepyrones |
| *FFUJ_05866* | PKS type III | 3.3 | -0.3 | 1.8 | -0.4 | 1.5 |  |
| *FFUJ_08113* | NRPS4 | 1.7 | 4.4 | 2.2 | -0.4 | 1.7 |  |
| *FFUJ_10736* | NRPS6 | 1.9 | -0.2 | -3.3 | -1.8 | 7.1 | Fusarinine |
| *FFUJ_10934* | NRPS11 | -3.3 | -0.7 | -1.0 | -0.2 | -6.4 |  |
| *FFUJ_14790* | NRPS12 | -1.0 | 1.3 | -2.9 | 0.2 | 5.4 |  |
| *FFUJ_12008* | NRPS23 | 1.4 | -0.9 | 2.7 | -1.8 | -1.3 |  |
| *FFUJ_00003* | NRPS31 | -0.3 | 0.4 | -4.9 | 2.6 | 8.7 | Apicidin F |
| *FFUJ_02115* | NRPS34 | 0.3 | 0.9 | -1.4 | -3.2 | 12.2 | Fusaric acid |
| *FFUJ_00036* | STC1 | -2.1 | -2.3 | -0.3 | -0.2 | -2.1 | Germacrene D |
| *FFUJ_10353* | STC4 | 4.0 | 0.3 | 3.4 | -0.1 | 4.0 | Koraiol |
| *FFUJ_14336* | DTC1 | -7.4 | 0.3 | -1.2 | -0.5 | -7.4 | Gibberellic acids |
| *FFUJ_11802* | TeTC1 | 3.7 | 0.0 | 2.3 | -0.9 | 3.7 | Neurosporaxanthin |
| *FFUJ_14683* | DMATS3 | 2.4 | 0.3 | 1.5 | -0.2 | -0.6 |  |

**Table S3.** **Primer sequences used for the generation of deletion constructs and for the verification of their homologous integration.** Introduced overhangs required for yeast recombinational cloning are underlined.

| **Gene** | **Primer** | **Sequence 5’ 🡪 3’** |
| --- | --- | --- |
| *SET1*  (*FFUJ_02475*) | set1_5F | GTAACGCCAGGGTTTTCCCAGTCACGACGCTATGTCTGTATGCCAGATGGC |
|  | set1_5R | ATCCACTTAACGTTACTGAAATCTCCAACGAGTGTACACCTAACGGTTGGG |
|  | set1_3F | CTCCTTCAATATCATCTTCTGTCTCCGACACTACTATTTGGCACCACACCG |
|  | set1_3R | GCGGATAACAATTTCACACAGGAAACAGCCATAGTGACAGGATTCGATACGC |
|  | set1_5diag | CTTGAGAGGTCACCATGGC |
|  | set1_3diag | CTCTCATTCCTTACATTCGC |
|  | set1_WT_F | CACAGCGACAACCTGTAGTCC |
|  | set1_WT_R | GAATCTGCTATAGCGAAGCTGG |
| *KDM5*  (*FFUJ_13017*) | kdm5_5F | GTAACGCCAGGGTTTTCCCAGTCACGACGACAAGCTGGCAATGCTCAATGG |
|  | kdm5_5R | ATCCACTTAACGTTACTGAAATCTCCAACGTACGTCGACTTCATACTCGG |
|  | kdm5_3F | CTCCTTCAATATCATCTTCTGTCTCCGACGAATACCACACCCTACATGAGG |
|  | kdm5_3R | GCGGATAACAATTTCACACAGGAAACAGCGTAGTGGTTGCGCTTACTGACG |
|  | kdm5_5diag | TGTCAGACTTGTTCCCTTCG |
|  | kdm5_3diag | ACTTGGTTGATCGGTGGTCC |
|  | kdm5_WT_F | CTACTATCGTGACCAGATGC |
|  | kdm5_WT_R | TCTCGTATGTGAGCAAGACC |
| *ABA1*  (*FFUJ_00702*)  (*FFE2_00769*) | aba1_5F | GTAACGCCAGGGTTTTCCCAGTCACGACGGCAAAGATGCAGAAGAAGGTTGG |
|  | aba1_5R | ATCCACTTAACGTTACTGAAATCCATGGCAACAAGCAAGTGTTTGGC |
|  | aba1_3F | CTCCTTCAATATCATCTTCTGTCGAGATGGATCATGCATTTACGCG |
|  | aba1_3R | GCGGATAACAATTTCACACAGGAAACAGCAGTGCGTCCCTTCAATATCCAACCC |
|  | aba1_5diag | AGAGGCAGTCGATGCTGAGGAGG |
|  | aba1_3diag | ACTAGGGACTGCTTCAGTAACCG |
|  | aba1_WT_F | CTGTCCTCACAACGCTATACACC |
|  | aba1_WT_R | CCAGGCTCCTTCTTCTCTTCCGC |
| *FLB2*  (*FFUJ_01507*) | flb2_5F | GTAACGCCAGGGTTTTCCCAGTCACGACGACGCGAGGTATAGCCAATTGGG |
|  | flb2_5R | ATCCACTTAACGTTACTGAAATCTCCAACTGGTCGCCTATGAGAGGTAGG |
|  | flb2_3F | CTCCTTCAATATCATCTTCTGTCTCCGACTCGAGTCAGGGGAGACGACAGC |
|  | flb2_3R | GCGGATAACAATTTCACACAGGAAACAGCAGGCTGCTTGTCATAAGAGCGG |
|  | flb2_5diag | CCACTGCTCCAACTGAGGTCGG |
|  | flb2_3diag | CAGCACGGTCGATTGGTGTGAGC |
|  | flb2_WT_F | GCTCAGAAGATGACAATGCTGCGG |
|  | flb2_WT_R | CCCATGTCTTCTGGTTCGTATCGC |
| *FLB3*  (*FFUJ_02801*) | flb3_5F | GTAACGCCAGGGTTTTCCCAGTCACGACGCATCTCAGACCCTCCACATC |
|  | flb3_5R | ATCCACTTAACGTTACTGAAATCTCCAACTGGTACGTGATAGTGGTGAT |
|  | flb3_3F | CTCCTTCAATATCATCTTCTGTCTCCGACTTCGCGCTTATTGCTGAGC |
|  | flb3_3R | GCGGATAACAATTTCACACAGGAAACAGCGGACGTTCTCCGATCAAA |
|  | flb3_5diag | GCAACAAGTCACCTACAGGCA |
|  | flb3_3diag | TCTCTACATTAGCTCCCTCG |
|  | flb3_WT_F | ATGACCATGACGCTCGACACCAC |
|  | flb3_WT_R | GTCGGAGTGATGGTCCTCAGAGCC |
| *FLB4*  (*FFUJ_12231*) | flb4_5F | GTAACGCCAGGGTTTTCCCAGTCACGACGTAGCTTCAACAAGGCAGCTCCG |
|  | flb4_5R | ATCCACTTAACGTTACTGAAATCTCCAACGTTGGGTGACGGTTACTGATTGG |
|  | flb4_3F | CTCCTTCAATATCATCTTCTGTCTCCGACGCAGTTATACGGTCGAAGATGGG |
|  | flb4_3R | GCGGATAACAATTTCACACAGGAAACAGCCCTACGGATGTAGTCTCGTACC |
|  | flb4_5diag | CTTTCAGGGTCTAGGTAATACGCC |
|  | flb4_3diag | ACCTCGATCAGTCGACCAATCG |
|  | flb4_WT_F | ATGGAACTTGTTCGCACGCATGG |
|  | flb4_WT_R | AACCTGGGTTGATACTCAGGCC |
| *FLB5*  (*FFUJ_04436*) | flb5_5F | GTAACGCCAGGGTTTTCCCAGTCACGACGACGCCATGCTTCAGACACGAGC |
|  | flb5_5R | ATCCACTTAACGTTACTGAAATCTCCAACTGAGAGTGAAGCTGCTGTGTGC |
|  | flb5_3F | CTCCTTCAATATCATCTTCTGTCTCCGACAGCCATCTCATGCTAAGACGGG |
|  | flb5_3R | GCGGATAACAATTTCACACAGGAAACAGCCTTGCGATCGAACTTCCTACGC |
|  | flb5_5diag | CTATGTCGAGGATCATGTCGCTTGC |
|  | flb5_3diag | GCTCAACATGTAAAGGAGAGCGACG |
|  | flb5_WT_F | GAAGATGCTGCACCAAACTGGG |
|  | flb5_WT_R | CTGGCAATGTCCTCGAGAGTGGC |
| *WET1*  (*FFUJ_07285*) | wet1_5F | GTAACGCCAGGGTTTTCCCAGTCACGACGGATGCTATGTGCTGCAAGAGGACG |
|  | wet1_5R | ATCCACTTAACGTTACTGAAATCTCCAACGCTGATGGGCGAGGATGCAATGG |
|  | wet1_3F | CTCCTTCAATATCATCTTCTGTCTCCGACGGTTCCTGGTTCCTGCATGAGG |
|  | wet1_3R | GCGGATAACAATTTCACACAGGAAACAGCAGAAGCTGTGGCAGTGGGGAGCC |
|  | wet1_5diag | ACCAGGGGCACCAGAAAGCAGCC |
|  | wet1_3diag | TCGATATCCGAGGTAGTACGCC |
|  | wet1_WT_F | CCAGGAGTCAAGACTGAACCTGGC |
|  | wet1_WT_R | CGCGCTTTGGTCTTAGAACTGCC |
| *CSM1*  (*FFUJ_07383*) | csm1_5F | GTAACGCCAGGGTTTTCCCAGTCACGACGTCGACATCGCTGTCAACGGG |
|  | csm1_5R | ATCCACTTAACGTTACTGAAATCTCCAACGTTCTCGATACGACCTGTCC |
|  | csm1_3F | CTCCTTCAATATCATCTTCTGTCTCCGACACTCTTCCCGCCACGGTGGG |
|  | csm1_3R | GCGGATAACAATTTCACACAGGAAACAGCTCATCAATGATGTCTCCGCC |
|  | csm1_5diag | CCAGCCGTGGCTCAAGGCCG |
|  | csm1_3diag | CACTGATCCCTCCAAAGCGC |
|  | csm1_WT_F | CACAACCCAGCTCTGGTGC |
|  | csm1_WT_R | GCGTAATGCAACCCGCAGG |
| *hphR* | hph_F | GTCGGAGACAGAAGATGATATTGAAGGAGC |
|  | hph_R | GTTGGAGATTTCAGTAACGTTAAGTGGAT |
|  | trpC_T | GGAATAGAGTAGATGCCGACCGG |
|  | trpC_P2 | GTGATCCGCCTGGACGACTAAACC |
| *natR* | nat1_hiF | CGGCGAGCAGGCGCTCTACATGAGC |

**Table S4. Primer sequences used for the generation and analysis of complementation, point-mutation and overexpression vectors.** Introduced overhangs required for yeast recombinational cloning are underlined.

| **Gene** | **Primer** | **Sequence 5’ 🡪 3’** |
| --- | --- | --- |
| *SET1*  (*FFUJ_02475*) | set1_c_R1 | GGTCTAGTAAAGACAGGAGCAGTTCCAG |
|  | set1_c_F2 | CTGGAACTGCTCCTGTCTTTACTAGACC |
|  | set1_c_R2 | CCCTAATCATACATCTTATCTACATACGTCAGTTGAGGAAGCCCTTAC |
|  | set1_mut_F | CGCGTTTCATCAACAAGAGTTGTGACCC |
|  | set1_mut_R | GGGTCACAACTCTTGTTGATGAAACGCG |
|  | set1_Seq1 | AGCTCATACAGTAGCACGG |
|  | set1_Seq2 | AATGATTAGGACGATGACGC |
|  | set1_Seq3 | TGGAAGAGCACCTCAAGAGG |
|  | set1_Seq4 | AGGATGACTTTGTGCCATGG |
|  | set1_Seq5 | CAAGTATGACGAGAAGCTCC |
|  | set1_Seq6 | GTTACGTACAAGACCTCAACG |
|  | set1_Seq7 | TCCAATTTCATCGCTACCTCG |
|  | set1_Seq8 | CTGCTCTCGGCAATTTCACCG |
|  | set1_Seq9 | GCTGTGGAGGTTGTTAGACGTG |
|  | set1_c_diag | CCGGTGTCGACCCAGATTTGG |
| *KDM5*  (*FFUJ_13017*) | OE_kdm5_F | CAACTCCATCACATCACAATCGATCCAAATGGTGTCAGTGCCAACTG |
|  | OE_kdm5_R | CCCTAATCATACATCTTATCTACATACGGGTGGTGGCTCTTCTCGCGG |
|  | kdm5_Seq1 | CGCACCTCGGTATCAATGGC |
|  | kdm5_Seq2 | GGGCATAGGCGTGTCGCC |
|  | kdm5_OE_diag | GGAATGGTCGCAAGACTCGC |
| *Tgluc* | BcGlu_Term_F2 | GCGGCCGCTTAGCGTATGTAGATAAGATGTATG |
|  | Tgluc_Nat1_R | CCACTTAACGTTACTGAAATCTCCAACATCTTGTTGGGGGGAAGGGGT |
|  | Tgluc_Seq_R2 | CCGCCCTCTTTTGTCTTCCGC |
| *natR* | nat1_R1 | CAGTGCCTCGATGGCCTCGGCGTC |
| *PoliC* | PoliC_Seq_F2 | GGGAGACGTATTTAGGTGCTAGGG |

**Table S5. Primer sequences for analysing relative expression (qPCR) and ChIP-qPCR (5’ChIP).** Reference genes for relative expression: *GMT*, GDP mannose transporter gene; *RAC*, related actin gene; *UBI*, ubiquitin gene.

| **Gene** | **Primer** | **Sequence 5’ 🡪 3’** |
| --- | --- | --- |
| *CPS/KS*  (*FFUJ_14336*) | CPS/KS_5’ChIP_F | TCAGACGCAATGTTGATATCTCGC |
|  | CPS/KS_5’ChIP_R | AGGTGTGTTTCATACGTACGGC |
| *P450-1*  (*FFUJ_14333*) | P450-1_5’ChIP_F | TGGCGAACTGTCCAACGTCC |
|  | P450-1_5’ChIP_R | ACCTCGTAGGCGTACTTTGGC |
| *P450-2*  (*FFUJ_14334*) | P450-2_5’ChIP_F | TGAGACTACGGCTTCTTCC |
|  | P450-2_5’ChIP_R | AGACGAATATGGCGATGTAGA |
| *P450-4*  (*FFUJ_14332*) | P450-4_5’ChIP_F | AGCATGAACAGTACCAGCC |
|  | P450-4_5’ChIP_R | AAGAGGACGATAGGACATGG |
| *ABA1*  (*FFUJ_00702*) | aba1_5’ChIP_F | CTGCATTCGTAGAGTCCTCAAAGGC |
|  | aba1_5’ChIP_R | GTGAGGACAGCACTGGCCTCG |
|  | aba1_qPCR_F | GGATGGTCTGCCAGCACTGG |
|  | aba1_qPCR_R | GTCTTCGCTTCATTGGCCTGGG |
| *FLB3*  (*FFUJ_02801*) | flb3_5’ChIP_F | CCCTCTACTACGCTTCTGCCCAC |
|  | flb3_5’ChIP_R | GCGCTGAATAGTCGAAATTCAACG |
|  | flb3_qPCR_F | CATGATGGGCCAGTTCAGCTCC |
|  | flb3_qPCR_R | TCCTCAGAGCCAGCCTCAGAGC |
| *FLB4*  (*FFUJ_12231*) | flb4_5’ChIP_F | CGTGAAACATCGTCACCAAGAGG |
|  | flb4_5’ChIP_R | TCCTCATATTGTGACCAAGGGCC |
|  | flb4_qPCR_F | TACCACCTCTGCGTCACTCAGGC |
|  | flb4_qPCR_R | AGATGCATCCTGGAATCTTGGGC |
| *WET1*  (*FFUJ_07285*) | wet1_qPCR_F | TCTCCTAATGACAGGTGTCGCGC |
|  | wet1_qPCR_R | ATGCAAAGCCTTGTTCCATCAGC |
| *GMT*  (*FFUJ_07710*) | FGMTRTPCRFW | CGGGCCATTCTCTATTCTTTC |
|  | FGMTRTPCRRV | ATGCTGTGATGGCAACAATG |
| *RAC*  (*FFUJ_05652*) | FRACRTPCRFW | GAGAACGAGCGTGTCTTGATTGAGCC |
|  | FRACRTPCRRV | TTTCCTCCGCAGAATGAAGAAGGACTC |
| *UBI*  (*FFUJ_08398*) | FUBRTPCRFW | CCAACCCTGACGATCCTCTTGTGC |
|  | FUBRTPCRRV | TACTTTCGAGTCCACTCCCGAGCTG |
